# Collection and Characterization of Wood Decay Fungal Strains for Developing Pure Mycelium Mats

**DOI:** 10.3390/jof7121008

**Published:** 2021-11-25

**Authors:** Marco Cartabia, Carolina Elena Girometta, Chiara Milanese, Rebecca Michela Baiguera, Simone Buratti, Diego Savio Branciforti, Dhanalakshmi Vadivel, Alessandro Girella, Stefano Babbini, Elena Savino, Daniele Dondi

**Affiliations:** 1 Department of Earth and Environmental Sciences, University of Pavia, 27100 Pavia, Italy; marco.cartabia01@universitadipavia.it (M.C.); rebeccamichela.baiguera01@universitadipavia.it (R.M.B.); simone.buratti01@universitadipavia.it (S.B.); elena.savino@unipv.it (E.S.); 2 MOGU S.r.l., Via S. Francesco 62, 21020 Inarzo, Italy; sb@mogu.bio; 3 Department of Chemistry, University of Pavia, 27100 Pavia, Italy; chiara.milanese@unipv.it (C.M.); diegosavio.branciforti@gmail.com (D.S.B.); dhanavadivel87@gmail.com (D.V.); alessandro.girella@unipv.it (A.G.); daniele.dondi@unipv.it (D.D.)

**Keywords:** wood decay fungi (WDF), mycelium-based material, fungal strain collection, scanning electron microscopy (SEM), thermogravimetric analysis (TGA)

## Abstract

Wood decay fungi (WDF) seem to be particularly suitable for developing myco-materials due to their mycelial texture, ease of cultivation, and lack of sporification. This study focused on a collection of WDF strains that were later used to develop mycelium mats of leather-like materials. Twenty-one WDF strains were chosen based on the color, homogeneity, and consistency of the mycelia. The growth rate of each strain was measured. To improve the consistency and thickness of the mats, an exclusive method (newly patented) was developed. The obtained materials and the corresponding pure mycelia grown in liquid culture were analyzed by both thermogravimetric analysis (TGA) and scanning electron microscopy (SEM) to evaluate the principal components and texture. TGA provided a semi-quantitative indication on the mycelia and mat composition, but it was hardly able to discriminate differences in the production process (liquid culture versus patented method). SEM provided keen insight on the mycelial microstructure as well as that of the mat without considering the composition; however, it was able to determine the hyphae and porosity dimensions. Although not exhaustive, TGA and SEM are complementary methods that can be used to characterize fungal strains based on their desirable features for various applications in bio-based materials. Taking all of the results into account, the *Fomitopsis iberica* strain seems to be the most suitable for the development of leather-like materials.

## 1. Introduction

Wood decay fungi (WDF) have the fundamental role of degrading the components of plant cell wall (cellulose, hemicellulose, and lignin), displaying different selectivity towards each one depending on the species, developmental stage, and environmental conditions [1,2]. Wood decay species in Basidiomycota (WDB) report the greatest spectrum of degradation patterns, which are either enzyme-based or just enzyme-primed. Moreover, many WDB are easily isolated in their dykariotic stage, which enables the mycelium to develop certain morphological features similar to those of the basidiome in culture [3,4].

More and more WDF are being studied to evaluate their potential applications in different fields: pharmacology, medicine and nutraceutics, enzyme production, cosmetics, and the biosorption and biodegradation of persistent organic pollutants [5,6,7,8,9,10]. Another emerging application that involves the use of WDF concerns bio-based materials. Increasing attention has been paid to this specific aspect of WDF all around the world in recent decades. In fact, plastic pollution is quickly becoming one of the most critical global problems in the modern world [11,12,13]. Plastic polymers are very versatile materials that cannot be totally eliminated. On the other hand, research into alternative sources that are able to create similar materials is a complementary strategy for the replacement of traditional plastics in cases where they are not indispensable.

Fungi are promising candidates thanks to the natural structure of their network, which is formed by the hyphae that are organized in the mycelium [14,15,16,17]. Mycelium technology has been attracting increasing attention in material sciences and in the design sector because fungal materials can be used for thermal/acoustic insulation and packaging, and in the design sector, they can replace already existing and polluting materials or can provide novel materials.

Currently, two main families of mycelium-based materials (myco-materials) are available: bio-composites and pure materials. In any case, the properties of myco-materials depend on the substrate, fungal species, and growth conditions. Up until to now, only few species of WDF have been tested as bio-fabricated materials, but hundreds of potentially suitable species grow in the world as well as in Italy [18,19]. Most of them have not been tested yet [16].

Mycelium-based composites result from the growth of filamentous fungi on organic materials such as agricultural waste streams. These novel bio-based materials represent a promising alternative for product design and manufacturing both in terms of sustainable processes and circular lifespan [20,21].

Pure fungal materials mainly consist of mycelium [20]. These materials are either the result of the complete degradation of the substrate or can be obtained by removing the fungal mat from the surface of a liquid or solid substrate [22].

The properties of myco-materials depend on the substrate, fungal species (i.e., fungal strategy), and growth conditions.

In recent years, the textile and fashion design industries have placed a great deal of emphasis on the development of new sustainable and compostable materials that are able to be obtained using environmentally friendly, non-pollutant processes. The production of fungal mats for textile applications promises more sustainable and compostable alternatives to the soft materials that are currently on the market, especially those from the leather sector [22,23].

Leather production has been increasingly raising ethical issues about animal welfare as well as the negative impact of leather production on the environment. The leather industry consumes substantial amounts of natural resources and uses toxic, persistent chemicals that are used for the processing of the skins [24,25]. Leather-like materials are currently made of polyvinyl chloride (PVC) and polyurethane (PU), but they are of environmental concern, at least in terms of their production/degradation processes. To produce animal-free and sustainable soft materials with leather-like properties, materials based on renewable feedstock have been proposed as alternatives to cotton or petroleum-based fibres; examples include pineapple cellulose (Piñatex), grape pomace (Vegeatex), orange fibre, and palm leaf products.

The aim of this study is to collect wood decay fungal strains that are suitable for the development of mycelium mats that are able to be used as a leather-like fashion design prototype. In the present research, differences in the texture and cell wall components of different mycelium species have been investigated through scanning electron microscopy (SEM) and thermogravimetric analysis (TGA) in order to identify certain possible functional characteristics of fungal strains that can be used as myco-materials.

Both SEM and TGA are increasingly being applied in various contexts and have proven to be useful tools that can be used to understand t the morphology and the essential components of a material, regardless of whether that material is artificial or biogenic [26,27]. Thermogravimetric analysis is a characterization technique that also allows the investigation of plant biomass constituents. It consists of a pyrolysis or combustion process that records mass losses as a function of temperature [28,29]. Pyrolysis enables the molecular decomposition of an organic material by heating and breaking chemical bonds, leading to the formation of simpler molecules. [30]. According to a temperature gradient, heat is applied up to a maximum of 700–900 °C in the presence of an inert gas [31]. Popescu et al. (2010) [32] used this technique to study the physical properties of wood by obtaining information on the interaction between cellulose, lignin, and hemicellulose. TGA has already been used to characterize the major constituents of the fungal cell wall (in particular β-glucans and chitin) [26].

## 2. Material and Methods

### 2.1. Collection of Wood Decay Fungal Strains

Many wooded areas in the north of Italy (Lombardia and Piemonte Regions) have been examined for samples that are suitable for the isolation of WDF strains to be taken. Different habitats (moorland, thermophilous broadleaf forest, riparian forest, mixed coniferous forest, ancient larch forest, and Mediterranean scrub) were explored and only basidiome that were actively growing were collected.

Basidiome identification was performed through dichotomous keys based on macro and micro-morphology [33,34]. The taxonomy check relied on Mycobank (www.mycobank.org; accessed on 13 November 2021). Whenever possible, specimens were added to the *Herbarium Universitatis Ticinensis* (DSTA, University of Pavia, Italy) or to the *herbarium* of Associazione Micologica Bresadola (AMB)—Varese section (Italy).

The standard protocol for mycelium isolation from the wild basidiomes [3,4,35] was slightly modified to the following procedure: To avoid breaking the basidiomes, only little pieces were collected from each sample. When the basidiome was too thin or small or compromised, pieces of wood that had been colonized by the fungus were collected. Subsequently, they were put in an aluminium sheet and were closed in a plastic box to allow a slightly humid environment suitable for the mycelium regrowth was established for few days. Then, three to six little portions of fresh and growing mycelium were taken from the basidiome under sterile conditions and were placed into a Petri plate containing 2% malt extract agar (MEA) (Biokar diagnostics, Allonne, France and VWR Chemicals, Milano, Italy) + 6 mL/L H_2_O_2_ (3%) [36]. When the colony reached a proper size (around 2 cm of diameter), a piece was transferred to an MEA 2% plate.

To maintain each strain in pure culture, 20 paper filter discs (5 mm diameter) were cut, sterilized, and disposed circularly into a 2% MEA plate. A plug of actively growing mycelium was put in the center, and after 7–15 days, the mycelium had spread to reach all of the paper discs [37]. For each strain, four discs were put in a sterilized 1.8 mL autoclavable polypropylene (PP) vial tube together with 1 mL of demineralized and sterilized water, and the tube was then sealed with parafilm. A minimum of five tubes per strain were stored in the dark at 4 °C, forming the MOGU S.r.l. (MOGU’s Fungal Strain Collection—MFSC) fungal research collection. Moreover, each WDF strain was maintained through various means, including storage at −80 °C in MicUNIPV, the Research Culture Collection of University of Pavia (Italy). The protocol for cryopreservation proposed by Homolka et al. 2014 was followed, with a few slight modifications [38]. After reaching optimal growth in liquid culture (2% ME), each mycelium was transferred to a 10 mL tube containing a 15% glycerol solution. The solution was homogenized through vortexing for 30 s at 3000 rpm. Then, 1 mL of the suspension was placed in 1.5 mL sterile cryotubes. For each fungal strain, four copies were stored at −80 °C.

### 2.2. Molecular Identification

Before including the strains in a database, their specific identification was confirmed by sequencing the ITS rDNA, which is generally recognized to be the major barcoding region for fungi [39,40]. Fungal strains were grown in flasks with 50 mL 2% malt extract and were incubated at 24 °C for 15 days in the dark in static. The mycelium was harvested and lyophilized in order to favor sample homogenization, as indicated by the kit manufacturer instructions for DNA extraction. DNA extraction was performed using a Macherey-Nagel Nucleospin plant II extraction kit. The ITS region was amplified using ITS1-ITS4 primers and a Dream Taq Mastermix (Promega, Milano, Italy).

In order to verify the DNA extraction and DNA amplification results, an electrophoretic run (75 V for 5 min, 100 V for 25 min) was performed on 1% agarose gel. SYBR Safe-DNA Gel Stain (Invitrogen, Waltham, MA, USA) was used as an intercalant, and BlueJuice (Invitrogen) was used as a gel loading buffer. Electrophoresis images were generated by Gel Doc (Biorad, Berkeley, CA, USA).

The purification of the post-PCR products was performed by adding ExoSAP-IT™ (Applied Biosystems, Foster City, CA, USA) in the following ratio: 5 µL of a post-PCR reaction product/2 µL of ExoSAP-IT™. The thermal protocol (Thermofisher, Waltham, MA, USA) included 15 min at 37 °C to degrade the remaining primers and nucleotides followed by 15 min at 80 °C to deactivate the ExoSAP-IT™ reagent.

DNA sequencing was performed by Macrogen Europe B.V. (Amsterdam, The Netherlands). The sequences were analyzed with the Sequencher 5.0 Demo software, and they were then matched with the sequences selected in different open repositories by means of MycoBank Molecular ID [41] and NCBI Nucleotide BLAST [42].

### 2.3. Choice of Fungal Strains

Among the strains that were successfully isolated, 21 strains (belonging to 20 species) were chosen based on the characteristics of each mycelium (Table 1). Most strains are well known for enzyme production and were less known for their application in bio-based materials. Species such as *Fomitopsis iberica*, *Neofavolus alveolaris*, and *Terana caerulea* not known for either enzyme production or for use in bio-based materials. The culture characteristics that were observed for every species were the color (white to cream or yellowish to brown or multicolor), the colony reverse (unchanged, bleached, darker), the general aspects of the mycelia (consistency of the mat), the behavior of the aerial and submerged hyphae, the presence of primordia or exudates (if any), and any smell in the event that it significantly characterized the mat production process.

Methods and terminology for the observation and description of mycelial features were retrieved from the literature and are reported in Table 1.

### 2.4. Fungal Growth under Different Conditions

Fungal growth and consistency are among the most influencing parameters for the production of a bio-based material [47]. In order to evaluate growth rate and mycelium consistency, two different procedures were conducted:

(a) Fungal strains growth rate

Even if many parameters can influence fungal growth, a standard approach using Petri plates (diameter 9 cm) on 2% MEA was adopted to evaluate the growth rate of the 21 strains [3]. In order to maximize the space available for radial growth, a mycelium plug (upper surface about 1 cm^2^) drawn from actively growing colonies (10 days old) was inoculated at the edge of the Petri plate (with the mycelium in direct contact with the MEA surface) and was incubated at 25 °C in the dark until the full colonization of the plate itself. The radius of the mycelium on the MEA medium was measured day by day for all of the strains by using a ruler until the edge of the Petri plate was reached, i.e., total coverage of the plate. The experiment was conducted in triplicate for each strain. The growth rate (mm day^−1^) of each strain was calculated at day 7 after inoculation as an average on three replicates.

(b) Method of producing fungal mats and materials made therefrom (patented)

In order to increase consistency and strength of the mycelium mat and the thickness of the hyphae, the method described hereafter has been developed and patented by MOGU S.r.l.: patent 102018000010869 issued by MISE (Ministero dello Sviluppo Economico) on 6 June 2020 [48] https://patents.google.com/patent/WO2020115690A1/en?oq=IT201800010869A1 (accessed on 13 November 2021).

**Figure 1:**
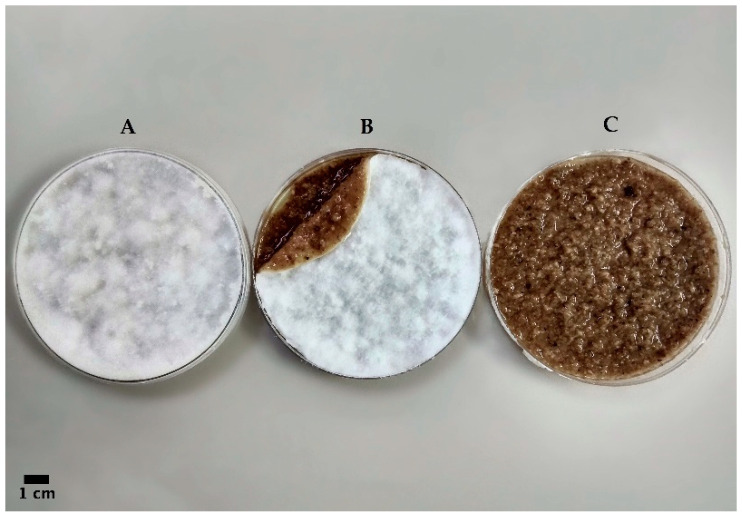
Fresh mycelial mat of *Fomes fomentarius* (8) obtained through patented method after 3 weeks growth in Petri plates: (**A**) the Petri after the 3-week growing phase; (**B**) a Petri where the removable mycelium mat that regrows on top of the slurry is visible; and (**C**) the remaining substrate after the removal of the mycelium mat.

**Figure 2:**
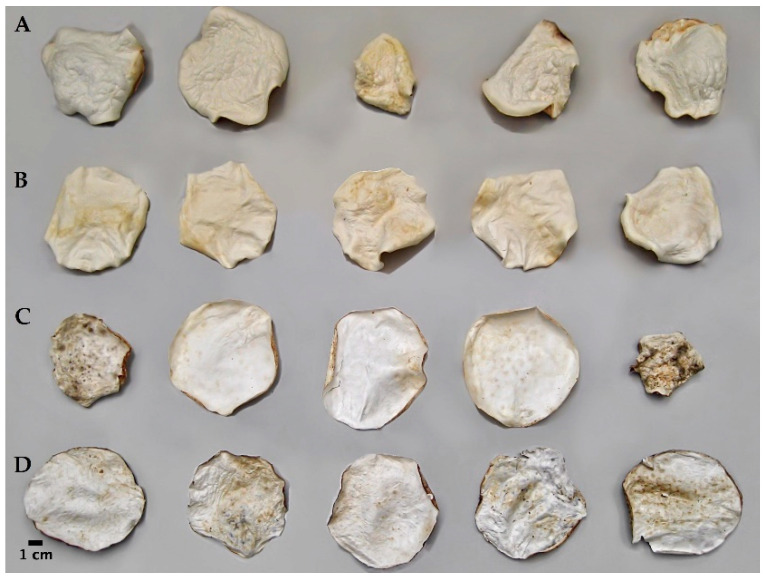
Dry mycelial mats obtained by Mogu’s patented method after 3 weeks of growth in Petri plates; five repetitions were conducted per strain: (**A**) *Fomitopsis iberica* (10), (**B**) *Daedaleopsis confragosa* (6), (**C**) *Coriolopsis gallica* (3), and (**D**) *Terana caerulea* (19).

At first, sterilized millet grains were used as a substrate in spawn bags with a microfilter to allow mycelium colonization for 28 days. Then, the resulting fungus–substrate complex (colonized millet) was blended, and the right amount of water was added in order to produce a creamy mixture that was poured into flat molds (9 cm diameter Petri plates) for 21 days to re-start the colonization process. Finally, the mycelial mat that had developed on the upper surface was removed (Figure 1). The dry weight (50 °C, 24 h) was recorded from the final mat after washing (Figure 2).

### 2.5. Thermogravimetric Analysis (TGA) on Mycelium Mats of the 21 Selected Strains Grown on Liquid Culture and Using Mogu’s Patented Method

The mycelium mats, which were obtained through the use of the slurry method, and the corresponding pure mycelia grown in liquid culture were analyzed by both thermogravimetric analysis (TGA) and scanning electron microscopy (SEM) in order to determine the functional characteristics in fungal strains that were suitable for their possible use in the context of myco-materials. All of the 21 selected strains in the present study were examined. 

Mycelium disks were obtained in 100 mL flasks containing 50 mL of 2% ME broth and were incubated in static at 25 °C in the dark for 21 days, and we assumed that growth had ceased for all of the strains after this period of time. Floating mycelial disks were rinsed with water to eliminate any remaining broth residues. The disks were then dried at 35 °C for 24 h to obtain the dry biomass since TGA requires the sample weight to be standardized and is highly biased by moisture [26]. 

TGA measurements were conducted using a TGA Q5000 instrument (TA Instruments, New Castle, DE, USA). An amount of up to 5 mg of each sample was weighted. The samples were heated from 25 °C to 900 °C at a scanning rate of 20 °C min^−1^ under nitrogen flux (25 mL min^−1^). The standards for β-glucan and chitin (both from Sigma Aldrich, St. Louis, MO, USA) as well as those for millet seeds were analyzed in the same conditions, allowing them to be compared with the fungal mass loss at different temperatures.

The thermogravimetric measurements are shown together with their first derivatives (DTGA) in order to better understand the decomposition temperature of the peaks (relative maximum of DTGA) and to locate the starting/ending of the decomposition (zeroing or flattening of DTGA).

### 2.6. Scanning Electron Microscopy (SEM) on Mycelium Mats Grown on Liquid Culture and Using Mogu’s Patented Method

Mycelium mats grown in liquid culture and through the described patented method were prepared as for TGA, as above mentioned.

SEM images were taken from a Zeiss EVO MA10 (Carl Zeiss, Oberkochen, Germany) scanning electron microscope equipped with a LaB_6_ emitter. Observations were performed in high vacuum mode through an Everhart–Thornley secondary electron detector at an accelerating voltage of 5 kV with a working distance between 8 and 9 mm. Dry samples were mounted onto aluminum stubs using double sided carbon adhesive tape and were then made to be electrically conductive through being coated in vacuum with a thin layer of Au.

In order to estimate the colony density from the SEM images, grayscale pictures were binarized, with white and black pixels being obtained on a bidimensional representation. The threshold tool Maximum Entropy from the ImageJ 1.53k software was used following the approach of Kapur et al. 1985 [49]. Maximum Entropy automatically defines an image-based threshold in order to optimize its contrast. Once the image had been binarized, the percentage of black pixels and white pixels was computed by the software itself. By convention and good proxy, the white pixels represented mycelium, while the black pixels represented inter-hyphae spaces (pores of the mycelial net). For each cultivation method that was used (static liquid culture and the patented method), three representative pictures per strain were selected, creating a total of 126 images. The picture selection criteria aimed to avoid any substrate or medium residue, thus maximizing the aerial hyphae area; furthermore, the pictures that were taken at the lowest and highest magnifications were discarded.

The hyphal thickness was determined using the measuring tool from the GNU Image Manipulation Program (GIMP) software through a proportion between length in pixels and the scale in µm, which is indicated on the photos that were obtained by SEM. For each strain, 15 measurements were taken.

Data from static liquid culture and patented method were compared using Wilcoxon test in R 4.0.3.

## 3. Results and Discussion

### 3.1. Wood Decay Fungal Strains

The field samplings successfully isolated 145 strains belonging to 55 genera and 87 species: we chose 21 strains (belonging to 20 species) to be examined for their potential in developing myco-materials.

In Table 2, the data regarding basidiome collection and strain code are reported.

The mycelium characteristics based on references are reported in Table 1. Concerning the three species for which no literature was available, *F. iberica* showed a mat that was white, downy to dense, thick, and quite homogeneous; notably, the colony grew from the edges of the Petri dish and continued to grow on the lid. *G. carnosum* has a mycelium that is thin, whitish when young and that is then olive-brown in the central part of the colony; it is also sometimes brown spotted; when the mycelium is transfered to other Petri dishes, it remains white for a longer amount of time and is homogeneous; it then becomes thicker and denser. The mat of *T. caerulea* appears to be white at first and then appears to be pigmented and dark blue and also appears to be quite homogeneous but thin and inconsistent; the pigmentation only occurs after storage at a low temperature (4 °C); otherwise it remains white.

Concerning the substrata, *Fomes fomentarius* (8), *Fomitiporia mediterranea* (9), and *Irpiciporus pachyodon* (15) grew on living trees, while all of the other taxa grew on dead wood. Despite such a categorization being increasingly resized and diminished, all of the examined species can be considered to be white rot agents, with the exception of *Fomitopsis iberica* and *Fomitopsis pinicola,* which are brown rot agents. 

The identities of all of the strains under examination were successfully confirmed by sequencing the ITS region, the method of which is reported in the Supplementary Materials. Particularly, the use of molecular analysis was required to confirm the cryptic species *Fomitiporia mediterranea*, which was otherwise indistinguishable from *Fomitiporia punctata sensu stricto* [45,50]. On the other hand, molecular identification must be critically addressed for species that are hardly able to be discriminated by the ITS region despite being quite easily discriminated by morphology and/or ecology, such as *D. confragosa* versus *D. tricolor* [51,52].

### 3.2. Fungal Strains Growth Rate

The recorded growth rates are reported in Table 3; the uncertainty of the random error (absolute uncertainties of the individual terms) was calculated according to Harris (2010) [53]. Despite the vast amount of literature that is devoted to WDF, true rational monographies that have focused on growth parameters in standard culture media are scarce [44,46]; thus, the main references concerning this specific aspect of WDF are Nobles (1948) [43] and Stalpers (1978) [3].

In this study, *B. adusta* (2) and *S. hirsutum* (18) showed a very high growth rate (11 mm day^−1^), confirming the literature data [3,42,43,53]. *A. biennis* (1) and *I. lacteus* (14) also showed high growth rate (>9 mm day^−1^). The strains of *C. gallica* (3–4) were similar and were reported to be 8 mm day^−1^. 

On the contrary, *G. lucidum* (13) reported the lowest growth rate; this is surprising since *G. lucidum* is usually considered to be a fast-growing species [54]. However, as highlighted by Dresch et al. (2015) [47], the variability among strains is not negligible. *T. caerulea* (19) and *D. confragosa* (6) also reported very low growth rates. *D. confragosa* and *D. tricolor*, whose basidiomes are easily discriminated by morphology, record different growth rates even though the two species are hardly recognized by standard molecular markers (e.g., ITS). This is why some authors consider *D. tricolor* as a variety of *D. confragosa* [51,52]. Marković et al. (2013) [55] actually dealt with *D. tricolor* and reported a higher growth rate than the one in the present study under the same cultural conditions.

A low growth rate was recorded *F. mediterranea* (9); this species was only proposed to be an independent taxon in 2002, as its growth rates can be hardly compared with references to *F. punctata,* with the exception of literature clearly referring to *F. mediterranea* itself [56].

### 3.3. Strains Cultivation Using Patented Method

**Figure 3:**
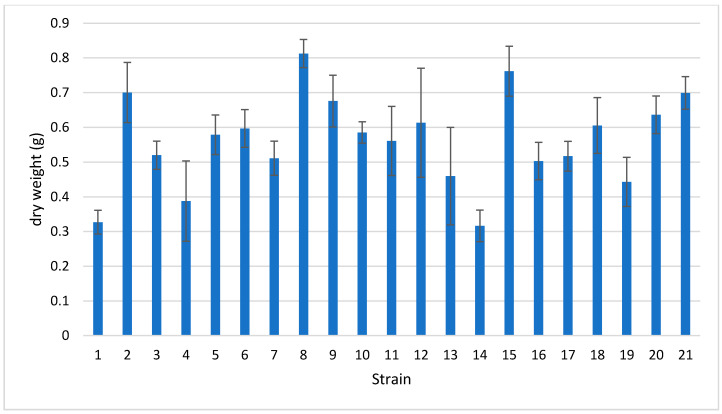
Average dry weight of mycelial mats obtained using the patented method (three repetitions each). The bars represent the standard error.

Among the 21 strains that were tested, some were unable to re-grow homogenous mats in the Petri plates. All of the obtained mats were dried at 50 °C for 24 h and were weighed (Figure 3).

*F. fomentarius* (8) and *I. pachyodon* (15) recorded the first and second highest dry weights, respectively, which is consistent with their compact mats. *B. adusta* (2) recorded the third highest dry weight despite the mat appearing fluffy and inconsistent.

All of the mats formed by the *Trametes* species were very fragile and inconsistent. *G. carnosum* (12) and *G. lucidum* (13) were unable to form a uniform mat. 

*F. pinicola* (11) formed a really dense mat but brittle and powdery. 

*A. biennis* (1), *C. gallica* (3 and 4), *C. trogii* (5), and *T. hirsuta* (20) formed a primordia of basidiomes on the upper surface of the mat after a few days, but the mat itself had not yet reached an acceptable consistency. However, a lack of primordia was observed in *A. biennis* (1) because the oxygen exchange was reduced due to the Petri plate being sealed; these conditions provided homogenous mats, similar to the one created by *F. fomentarius* (8). Thus, the mats from *A. biennis* (1) interestingly displayed an appreciable consistency despite its relatively low weight, as did the *I. lacteus* (14) mats.

*F. iberica* (10) and *F. mediterranea* (9) produced consistent and homogeneous mats that often also showed aerial mycelium; however, the latter species was very slow growing. 

As a whole, the dry weight of the mycelial mats was variably affected by the substrate residues (slurry residues) that were embedded in the mats themselves, and therefore, the mats were impossible to remove. This bias was less negligible when the mat was more consistent, i.e., in *F. fomentarius* (8) and *I. pachyodon* (15).

### 3.4. TGA on Mycelium Mats

TGA profiles reflect the different compositions of each mycelium (see Supplementary Material).

In order to better assess the differences between samples, the first derivatives of the TGA curves (DTGA) are considered in the discussion. The samples are compared to a growing material (millet) and with the main constituents of mycelium (chitin and beta glucans).

**Figure 4:**
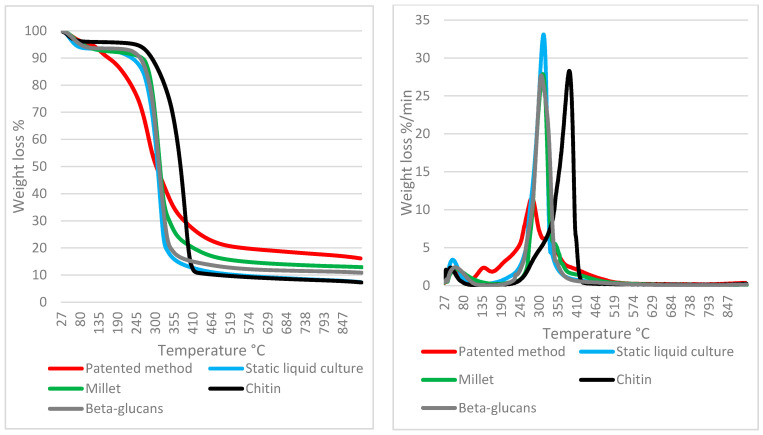
TGA (**left**) and DTGA (**right**) profiles of *Fomitopsis iberica* (10) (pure mycelium grown in liquid and mat grown on slurry) compared to reference materials.

**Figure 5:**
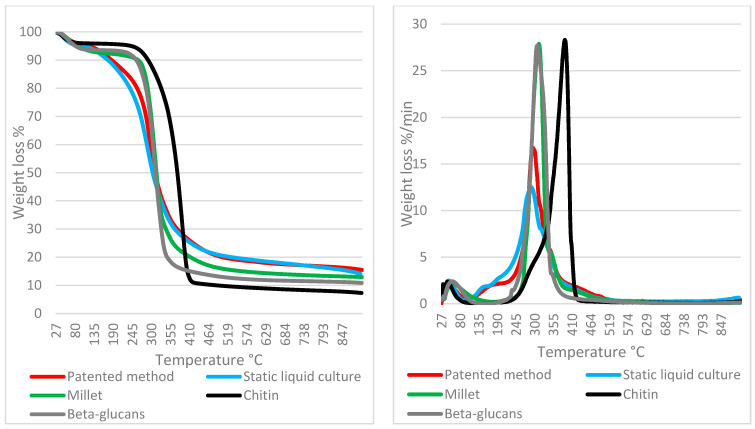
TGA (**left**) and DTGA (**right**) profiles of *Lenzites betulinus* (16) (pure mycelium grown in liquid and mat grown on slurry) compared to reference materials.

It is noteworthy that the mycelia that were obtained in the liquid culture using the patented method show profiles that are not comparable to each other in certain cases (e.g., strain 10), whereas in others, the respective profiles are similar (e.g., strain 16) (Figure 4 and Figure 5).

Taking the abovementioned strains as an example, the pure mycelium (strain 16) profile in Figure 5 almost overlaps with the slurry mat profile; the mass loss behaviors of both strains are both visibly driven by the component decomposition of β-glucan. However, the major difference between strain 16 and strain 10 is that the β-glucans in pure mycelium are much more developed in the latter (10), whereas the slurry mat is less variable in terms of its weight loss percentage.

β-glucans are also the major determinant in the millet profile since cereals are also rich in β-glucans.

**Figure 6:**
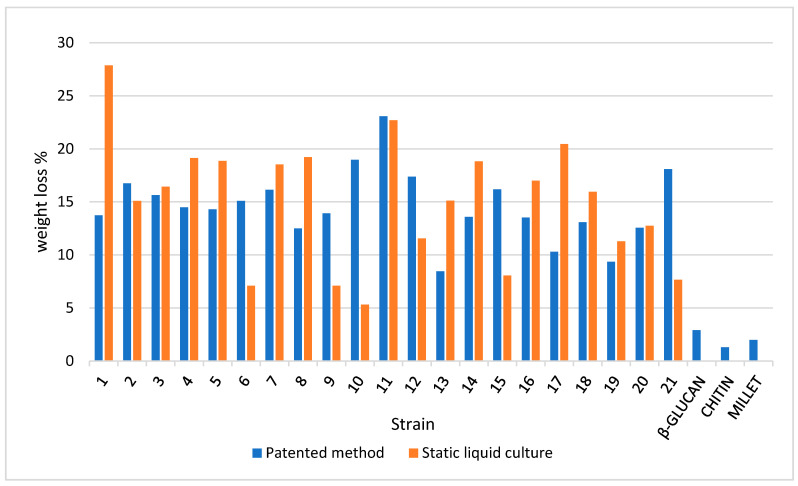
Weight loss percentage for the dry samples in the 125–250 °C temperature range (TGA output) in the mycelium (pure mycelium and slurry mat) and reference materials.

**Figure 7:**
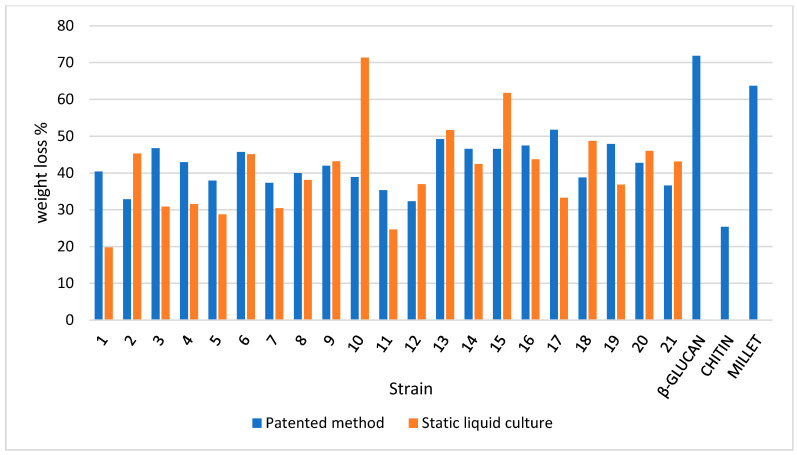
Weight loss percentage for the dry samples in the 250–350 °C temperature range (TGA output) in the mycelium (pure mycelium and slurry mat) and reference materials.

**Figure 8:**
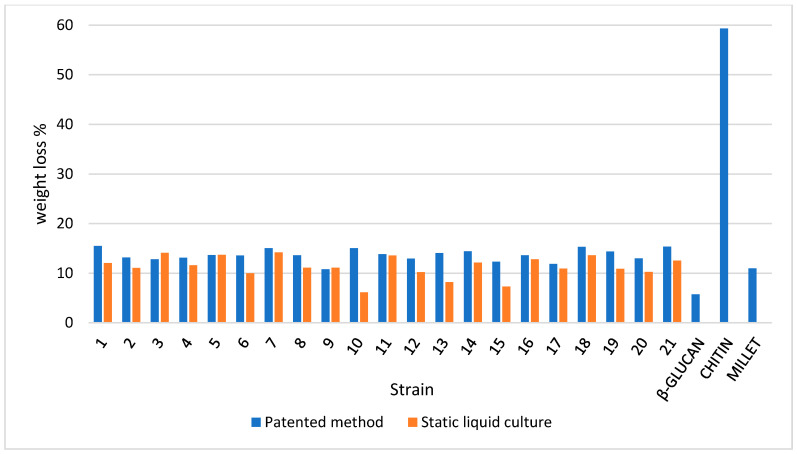
Weight loss percentage for the dry samples in the 350–500 °C temperature range (TGA output) in the mycelium (pure mycelium and slurry mat) and reference materials.

As expected, chitin is a minority component at this growth stage, but it is clearly shown by tailing when the temperature is over 350 °C, as was also the case in previous studies on wood decay mycelia [26]. Interestingly, the profiles of strain 16 and strain 10 suggest different behaviors: strain 16 displays a consistent β-glucan component in both the mycelia and slurry mat followed by moderate but overlapping tailing in the chitin degradation range; in strain 10, the pure mycelium is sharply focused on the β-glucan component, whereas the slurry mat developed less β-glucans but displayed a clear chitin tailing instead. Again, similar behaviors were suggested by the profiles discussed in Girometta et al. (2020) [26]. Notably, neither strain 16 nor 10 melanized; thus, the melanin variable can be excluded from the drivers of thermal behavior. Figure 6, Figure 7 and Figure 8 generalize the comments above to the whole strain spectrum that is under examination.

As clearly shown in Figure 6, a weight loss percentage in the range of 125–250 °C is different in terms of both the mycelium and slurry mat for most strains. More than 50% of the strains recorded higher losses in pure mycelium. Therefore, the slurry appears to be more focused on the production of β-glucans, with notable exceptions, such as strain 10, which was discussed above. This confirms that each species (as well strain, at intra-specific level) must be characterized before its application in biomaterials. The differences between pure mycelium and slurry mat behavior are attenuated when the percentage weight loss in the range of 250–350 °C as well as in the range of 350–500 °C is considered, with a few notable exceptions (e.g., strain 10).

### 3.5. SEM on Mycelium Mats Grown in Liquid Medium and Following Mogu’s Patented Method

The mycelia of all the 21 strains were observed by SEM. Microscopy was performed on both the mycelia grown in liquid culture and that had been grown using the patented method.

**Figure 9:**
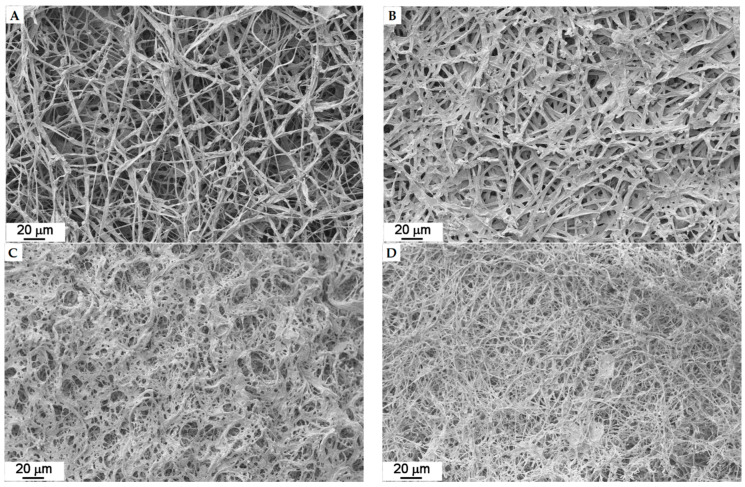
Different kinds of mycelia in strains: (**A**) *Coriolopsis trogii* (5), (**B**) *Irpex lacteus* (14), (**C**) *Daedaleopsis confragosa* (6), and (**D**) *Fomitopsis iberica* (10).

Concerning the images that were obtained from the pure mycelium, they show that the micro-structure is species-specific, i.e., each species has its own specific microstructure, and therefore, each texture is consequently very variable, forming loose to dense mycelium, as shown in Figure 9, where four examples are reported.

**Figure 10:**
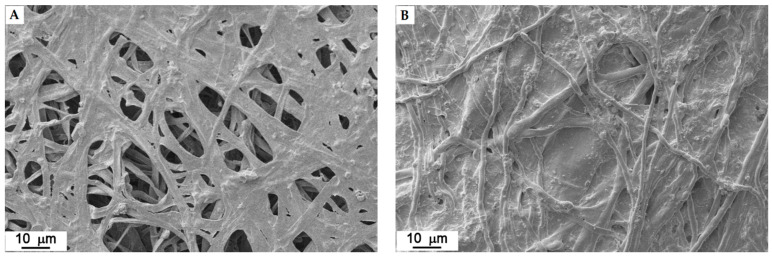
*Fomitopsis iberica* (10). Front (**A**) and back (**B**) of the mat obtained by patented method.

**Figure 11:**
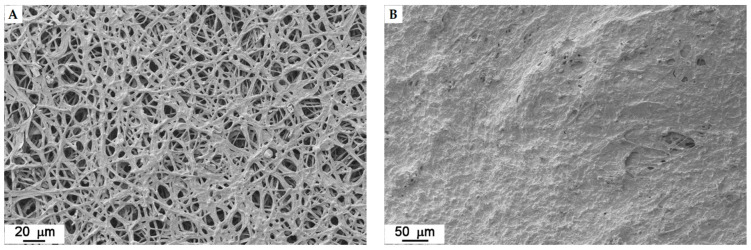
*Irpiciporus pachyodon* (15). Front (**A**) and back (**B**) of the mat obtained by patented method.

For each strain, the images of the mats that were obtained using the newly patented method are very similar to the ones that were produced by the mycelia obtained in liquid culture. The substrate residues are more evident in some strains than in others. The upper face (front) is always composed of a hairy (aerial) hyphae, while the bottom one (back) is always more compact and is flattened since it was in contact with the substrate (Figure 10 and Figure 11).

**Figure 12:**
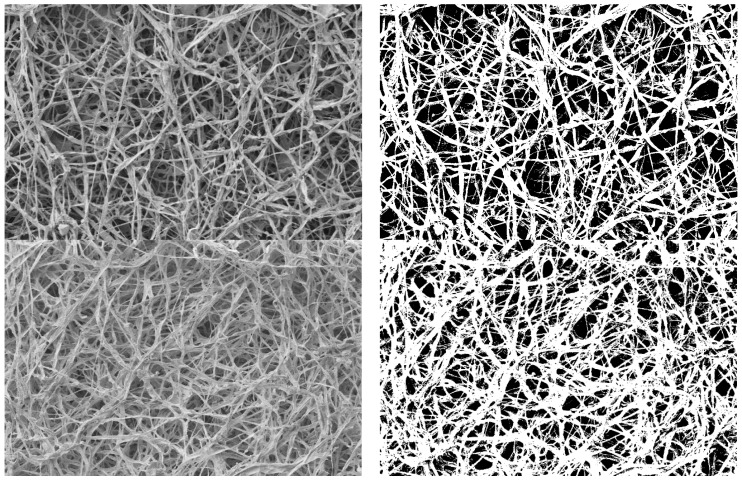
Examples of SEM images before (**left**) and after (**right**) binarization.

**Figure 13:**
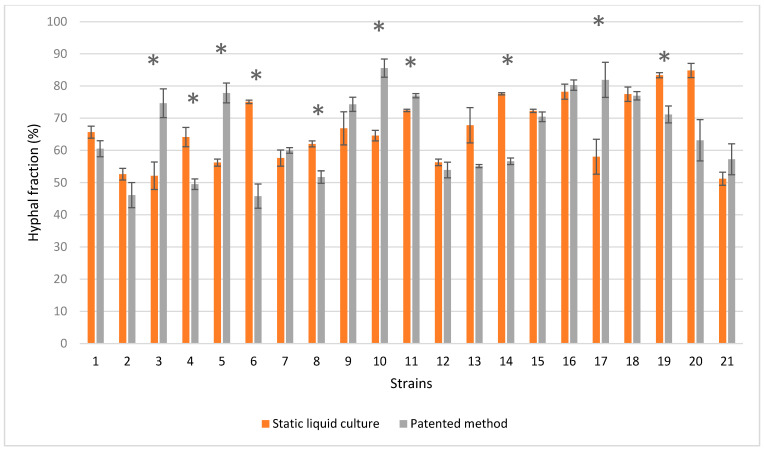
Average ± standard error of hyphal fraction obtained in liquid static culture and by patented method. Significantly different hyphal fractions are marked by * based on Wilcoxon’s test (* *p*-value < 0.05).

The binarization of the SEM images (Figure 12) was computed in order to quantify the concept of “dense” versus “loose” mycelium, i.e., a more or less compact mat. For each strain, the hyphal fraction (the projected area occupied by hyphae) was computed as the percentage of white pixels in the image versus the percentage of black pixels (i.e., the empty spaces). Results are shown in Figure 13.

The higher hyphal fraction was reported by *T. caerulea* (19) and *T. hirsuta* (20) in the static liquid culture, whereas it was reported by *F. iberica* (10), *L. betulinus* (16), and *N. alveolaris* (17) when the patented method was used. Strain by strain comparisons that were determined using Wilcoxon’s test revealed that 10 out of 21 strains developed a significantly different hyphal fraction when the cultivation method was changed (Figure 13): the hyphal fraction was particularly improved in the static liquid culture in *C. gallica* (4), *D. confragosa* (6), and *I. lacteus* (14), whereas the hyphal fraction was better in *C. gallica* (3), *C. trogii* (5), *F. iberica* (10), and *N. alveolaris* (17) when the patented method was used.

As a whole, the mycelium obtained by the patented method does not seem to produce a statistically different hyphal fraction than the ones obtained in a static liquid culture (Wilcoxon test: *p*-value > 0.05). 

**Figure 14:**
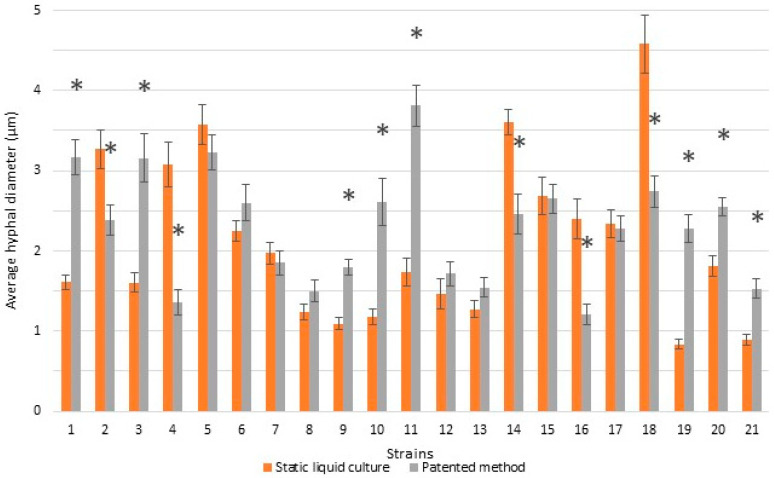
Average ± standard error of mycelium hyphal diameter obtained in liquid static culture and by patented method. Significantly different diameters are marked by * based on Wilcoxon’s test (* *p*-value < 0.05).

Moreover, the mean diameter of the hyphae was measured based on the SEM images, i.e., on a two-dimensional projection. The results are shown in Figure 14.

In static liquid culture, the higher hyphal diameter was reported by *C. trogii* (5), *I. lacteus* (14), and *S. hirustum* (18), but with patented method, it was reported by *A. biennis* (1), *C. trogii* (5), and *F. pinicola* (11).

Strain by strain tests revealed 13 out of 21 strains developed hyphae with a significantly different width when the mycelia were obtained from the liquid culture versus when obtained following the patented method (Figure 14): t *C. gallica* (4), and *S. hirustum* (18) showed significant improvements in the hyphal diameter when the static liquid culture was used, whereas with the patented method, hyphal diameter improvement was seen in *A. biennis* (1), *F. iberica* (10), and *F. pinicola* (11).

As a whole, the mycelium that was obtained by following the patented method seems to produce statistically different hyphae rather than those obtained in static liquid culture (Wilcoxon test: *p*-value < 0.05).

*C. gallica* was the only species to have two trains tested. The two strains both grew rapidly but behaved in opposite ways depending on whether they were grown in the static liquid culture or using the newly patented method. This indicates how important strain selection is when considering the production of bio-based materials.

**Figure 15:**
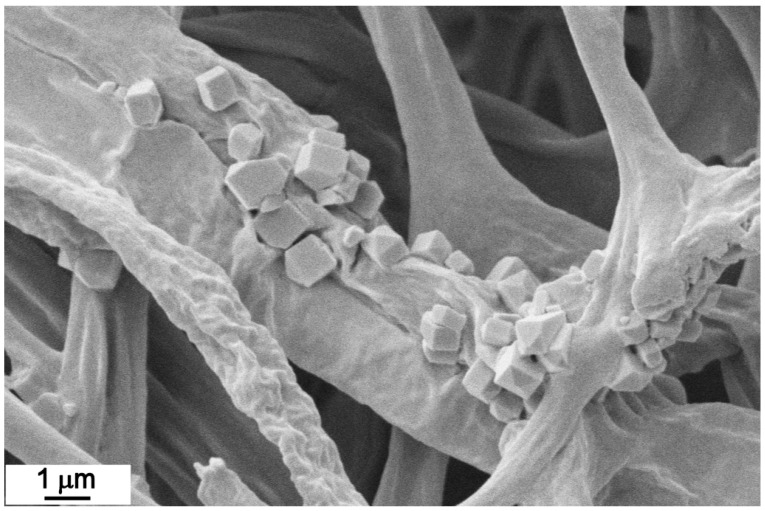
Typical oxalate crystals in *Fomes fomentarius* (8).

In the end, there are other additional components that could have affected the consistency and structure of the produced mats, e.g., the oxalate production observed in the *F. fomentarius* (8) and *L. betulinus* (16) mycelia (Figure 15). It is possible that the oxalates could cause the mat to be less uniform.

## 4. Conclusions

The structure and texture of each mycelium are highly variable due to species-specific and strain-specific features as well as to growth conditions. Consequently, mycelium characterization in material science requires an integrated, multi-focus approach in order to improve knowledge for next-process tuning.

The aim of the present work was to compare different species of wood decay fungi using two growth cultural methods. In particular, the characteristics that were determined for each mycelium were considered in the context of their use for the development of compact and resistant mats in the future.

Among the 21 strains that were examined, *A. biennis* (1), *B. adusta* (2), *I. lacteus* (14), and *S. hirsutum* (18) could be good choices for the development of myco-materials due to their high growth rate, but the mats that were obtained were light and fragile, meaning that they may not be suitable for the application of developing leather-like materials. 

TGA provides a good approximation on the composition of the cell wall; this enables each strain to be characterized shows the significant differences among the different strains. From a compositional perspective, the present study has also documented that TGA can point out differences depending on cultivation condition and the production process that is used to make the mat (namely static liquid culture versus the newly patented method). Composition is not the only determinant of mycelium mat features. This study remarks that SEM imaging is an excellent tool that is able to point out structural details and differences based on species and the mat production process.

SEM image processing revealed that *F. iberica* (10), *T. caerulea (19), and T. hirsuta* (20) produced mats with higher hyphal fractions, while *C. trogii* (5), *F. pinicola* (11), and *S. hirsutum* (18) showed the highest values in terms of their hyphal diameters.

The newly patented method improved the *F. iberica* (10) mats: TGA not only showed a different composition among the principal constituents of the hyphae (higher beta glucans fraction), but the analysis of the SEM images showed an improvement both in terms of the hyphal fraction and hyphal diameter.

In conclusion, TGA and SEM are complementary methods; despite SEM being more time-consuming and expensive and even though it requires remarkable personal skills, TGA is not capable of selecting strains that are appropriate for bio-based material applications on its own.

## Figures and Tables

**Table 1:** WDF chosen for developing mycelium mats.

Strain Code	Fungal Species	Mycelium Characteristics
1	*Abortiporus biennis* (Bull.) Singer	Colony white to cream, some felty parts; aerial hyphae often resembling skeletal one, submerged hyphae up to 7.5 µm wide. Primordia of sporophores (none of which reaches maturity) are easily formed [3].
2	*Bjerkandera adusta* (Willd.) P. Karst.	Mat white; at first silky becoming cottony-woolly, woolly floccose; advancing zone raised, reaching the lid of the Petri dish [3,43].
3	*Coriolopsis gallica* (Fr.) Ryvarden	Mat white mat at the beginning of its growth, then becomes olive-brownish and thin and crusty after 4–5 days. Aereal hyphae pigmented. Reverse creamed. Appressed to the margin, farinaceous to felty [3].
4	*Coriolopsis gallica* (Fr.) Ryvarden	
5	*Coriolopsis trogii* (Berk.) Domanski	Colony white to cream; marginal hyphae appressed, mat downy to felty; thin and almost transparent. It becomes fluffy and inconsistent [3].
6	*Daedaleopsis confragosa* (Bolton) J. Schröt.	Colony white darkening with age, presence of crustose areas hazel, “pinkish cinnamon” or “snuff brown”; mat downy to fine woolly, becoming felty; reverse darkened [3,43,44].
7	*Daedaleopsis tricolor* (Bull.) Bondartsev & Singer	Colony white, dense, downy—felty, homogeneous when young. After a few weeks of incubation, several primordia of sporophores (none of which reaches maturity) are formed [44].
8	*Fomes fomentarius* (L.) Fr.	Colony wthite to cream to chamois, reverse darker. Advancing zone raised with aerial mycelium uniform. Mat downy to cottony or woolly, then appressed and felty, relatively homogeneous. When it reaches maturity, the mycelium forms brown crusty leathery areas [3,43,44]
9	*Fomitiporia mediterranea* M. Fisch.	Mycelial cultures are cottony to woolly, with aerial hyphae that are yellowish to brownish; mat characterized by a sparse development of aerial hyphae that easily reach the lid of the Petri dish [45].
10	*Fomitopsis iberica* Melo & Ryvarden	References not available; see results 3.1
11	*Fomitopsis pinicola* (Sw.) P. Karst.	Mat white, heterogeneous, at first raised, cottony and woolly; usually uniform in appearance, sometimes forming scattered dots of more compact mycelium [3,43,44].
12	*Ganoderma carnosum* Pat.	References not available; see results 3.1
13	*Ganoderma lucidum* (Curtis) P. Karst.	Mat white with darker (brownish) zones, concentrically arranged, appressed, and powdery near the point of inoculation. Distal zone is ± homogeneous and felty, with small hyphal clusters [46]
14	*Irpex lacteus* (Fr.) Fr.	Mat white, downy to cottony and woolly floccose. Reverse bleached. Advanced zone raised. Some aerial hyphae with thickened walls [3].
15	*Irpiciporus pachyodon* (Pers.) Kotl. & Pouzar	Mat white, downy to cottony to felty with sometimes scattered dots of more compact mycelium. Aerial hyphae easily reaching the lid of the Petri dish [3].
16	*Lenzites betulinus* (L.) Pilát	Mat at first floccose and woolly, beoming patchy, with some areas raised, felty-woolly;intervening areas appressed, thin felty [47]
17	*Neofavolus alveolaris* (DC.) Sotome & T. Hatt.	Colony white to cream. Mat thick, dense and homogeneous at the beginning, then fluffy and woolly; also develops on the edges of the plate. Skeletal hyphae [3,44]
18	*Stereum hirsutum* (Willd.) Pers.	Cream to yellow to light brown with dark brown areas; mycelium heterogeneous, downy to felty areas alternating with translucent ones. The advancing zone is irregular, sinuous [3,44]
19	*Terana caerulea* (Schrad. ex Lam.) Kuntze	References not available; see results 3.1
20	*Trametes hirsuta* (Wulfen) Lloyd	Mat cottony to cottony and woolly and becoming (sub)felty and sometimes farinaceous, white to cream. Advancing zone appressed to raised. Reverse bleached. Most of skeletal hyphae are branched [3].
21	*Trametes suaveolens* (L.) Fr.	Mat white at first, later cream with orange tinges. Downy floccose, becoming woolly to subfelty. Advancing zone appressed to slightly raised. Odor strong, sweet [3,43].

**Table 2:** Data about basidiome and strain code of the WDF chosen for developing mycelium mats.

Code	Species	Basidiomes Sample Sites (Italy)	Date of Basidiomes Collection	Host Species	Basidiomes *Legit* and *Determinavit*	MFSC Code
1	*Abortiporus biennis*	Varese (VA)	24 November 2018	*Tilia cordata*	M. Cartabia	064-2018
2	*Bjerkandera adusta*	Albizzate (VA)	29 May 2019	*Populus alba*	M. Cartabia	101-2019
3	*Coriolopsis gallica*	Varese (VA)	30 March 2019	*Fraxinus excelsior*	M. Cartabia	086-2019
4	*Coriolopsis gallica*	Varese (VA)	23 June 2019	*Fagus sylvatica*	M. Cartabia	121-2019
5	*Coriolopsis trogii*	Cazzago Brabbia (VA)	12 June 2018	*Populus tremula*	M. Cartabia	027-2018
6	*Daedaleopsis confragosa*	Inarzo (VA)	29 August 2019	*Fraxinus excelsior*	M. Cartabia	155-2019
7	*Daedaleopsis tricolor*	Baceno (VB)	3 August 2019	*Prunus avium*	M. Cartabia	148-2019
8	*Fomes fomentarius*	Viterbo (VT)	2 December 2019	*Fagus sylvatica*	M. Cartabia	179-2019
9	*Fomitiporia mediterranea*	Varese (VA)	17 December 2018	*Fagus sylvatica*	M. Cartabia	079-2018
10	*Fomitopsis iberica*	Varese (VA)	8 June 2019	*Cedrus deodara*	M. Cartabia	104-2019
11	*Fomitopsis pinicola*	Valganna (VA)	22 June 2019	*Picea abies*	M. Cartabia	117-2019
12	*Ganoderma carnosum*	Varese (VA)	6 September 2019	*Picea abies*	M. Cartabia	161-2019
13	*Ganoderma lucidum*	Varese, (VA)	16 July 2019	*Quercus pubescens*	M. Cartabia	137-2019
14	*Irpex lacteus*	Cazzago Brabbia (VA)	7 December 2018	*Populus tremula*	M. Cartabia	076-2018
15	*Irpiciporus pachyodon*	Imperia (IM)	9 November 2019	*Quercus pubescens*	M. Cartabia	175-2019
16	*Lenzites betulinus*	Cittiglio (VA)	8 April 2019	*Betula pendula*	M. Cartabia	088-2019
17	*Neofavolus alveolaris*	Varese (VA)	3 May 2019	*Populus alba*	M. Cartabia	096-2019
18	*Stereum hirsutum*	Inarzo (VA)	5 December 2018	*Corylus avellana*	M. Cartabia	073-2018
19	*Terana caerulea*	Varese (VA)	2 November 2019	*Ostrya carpinifolia*	M. Cartabia	177-2019
20	*Trametes hirsuta*	Varese (VA)	25 November 2018	*Fagus sylvatica*	M. Cartabia	067-2018
21	*Trametes suaveolens*	Cazzago Brabbia (VA)	4 December 2018	*Salix alba*	M. Cartabia	070-2018

**Table 3:** Average growth rate calculated at day 7 after inoculation on the three replicates (random error ±2 mm).

Code	Species	MFSC Code	Average Growth Rate (mm day^−1^)
1	*Abortiporus biennis*	064-2018	9
2	*Bjerkandera adusta*	101-2019	11
3	*Coriolopsis gallica*	086-2019	8
4	*Coriolopsis gallica*	121-2019	8
5	*Coriolopsis trogii*	027-2018	6
6	*Daedaleopsis confragosa*	155-2019	4
7	*Daedaleopsis tricolor*	148-2019	7
8	*Fomes fomentarius*	179-2019	7
9	*Fomitiporia mediterranea*	079-2018	5
10	*Fomitopsis iberica*	104-2019	7
11	*Fomitopsis pinicola*	117-2019	6
12	*Ganoderma carnosum*	161-2019	6
13	*Ganoderma lucidum*	137-2019	2
14	*Irpex lacteus*	076-2018	10
15	*Irpiciporus pachyodon*	175-2019	5
16	*Lenzites betulinus*	088-2019	7
17	*Neofavolus alveolaris*	096-2019	7
18	*Stereum hirsutum*	073-2018	11
19	*Terana caerulea*	177-2019	4
20	*Trametes hirsuta*	067-2018	6
21	*Trametes suaveolens*	070-2018	7

## Data Availability

Data are available in the article and in the supplementary files.

## Supplementary Materials

The following are available online at https://www.mdpi.com/article/10.3390/jof7121008/s1, Figure S1: TGA (left) and DTGA (right) profiles of strain 1 (pure mycelium and slurry mat) compared to reference materials, Figure S2: TGA (left) and DTGA (right) profiles of strain 2 (pure mycelium and slurry mat) compared to reference materials, Figure S3: TGA (left) and DTGA (right) profiles of strain 3 (pure mycelium and slurry mat) compared to reference materials, Figure S4: TGA (left) and DTGA (right) profiles of strain 4 (pure mycelium and slurry mat) compared to reference materials, Figure S5: TGA (left) and DTGA (right) profiles of strain 5 (pure mycelium and slurry mat) compared to reference materials, Figure S6: TGA (left) and DTGA (right) profiles of strain 6 (pure mycelium and slurry mat) compared to reference materials, Figure S7: TGA (left) and DTGA (right) profiles of strain 7 (pure mycelium and slurry mat) compared to reference materials, Figure S8: TGA (left) and DTGA (right) profiles of strain 8 (pure mycelium and slurry mat) compared to reference materials, Figure S9: TGA (left) and DTGA (right) profiles of strain 9 (pure mycelium and slurry mat) compared to reference materials, Figure S10: TGA (left) and DTGA (right) profiles of strain 10 (pure mycelium and slurry mat) compared to reference materials, Figure S11: TGA (left) and DTGA (right) profiles of strain 11 (pure mycelium and slurry mat) compared to reference materials, Figure S12: TGA (left) and DTGA (right) profiles of strain 12 (pure mycelium and slurry mat) compared to reference materials, Figure S13: TGA (left) and DTGA (right) profiles of strain 13 (pure mycelium and slurry mat) compared to reference materials, Figure S14: TGA (left) and DTGA (right) profiles of strain 14 (pure mycelium and slurry mat) compared to reference materials, Figure S15: TGA (left) and DTGA (right) profiles of strain 15 (pure mycelium and slurry mat) compared to reference materials, Figure S16: TGA (left) and DTGA (right) profiles of strain 16 (pure mycelium and slurry mat) compared to reference materials, Figure S17: TGA (left) and DTGA (right) profiles of strain 17 (pure mycelium and slurry mat) compared to reference materials, Figure S18: TGA (left) and DTGA (right) profiles of strain 18 (pure mycelium and slurry mat) compared to reference materials, Figure S19: TGA (left) and DTGA (right) profiles of strain 19 (pure mycelium and slurry mat) compared to reference materials, Figure S20: TGA (left) and DTGA (right) profiles of strain 20 (pure mycelium and slurry mat) compared to reference materials, Figure S21: TGA (left) and DTGA (right) profiles of strain 21 (pure mycelium and slurry mat) compared to reference materials, Table S1. ITS sequences of the strains and main parameters on Mycobank Molecular ID and NCBI.
