# Supplementary material for: Collection and Characterization of Wood Decay Fungal Strains for Developing Pure Mycelium Mats
Source: J Fungi (Basel). 2021 Nov 25;7(12):1008. doi: 10.3390/jof7121008 (PMC8703653; doi:10.3390/jof7121008)
Supplement: Supplementary file 1 [file jof-07-01008-s001.zip › jof-1408627-supplementary.pdf]

Selection of wood decay fungal strains by TGA and SEM for developing pure mycelium mats

Cartabia Marco, Girometta Carolina Elena, Milanese Chiara, Baiguera Rebecca Michela, Buratti Simone, Branciforti Diego Savio, Dhanalakshmi Vadivel, Girella Alessandro, Babbini Stefano, Savino Elena, Dondi Daniele

Supplementary Material

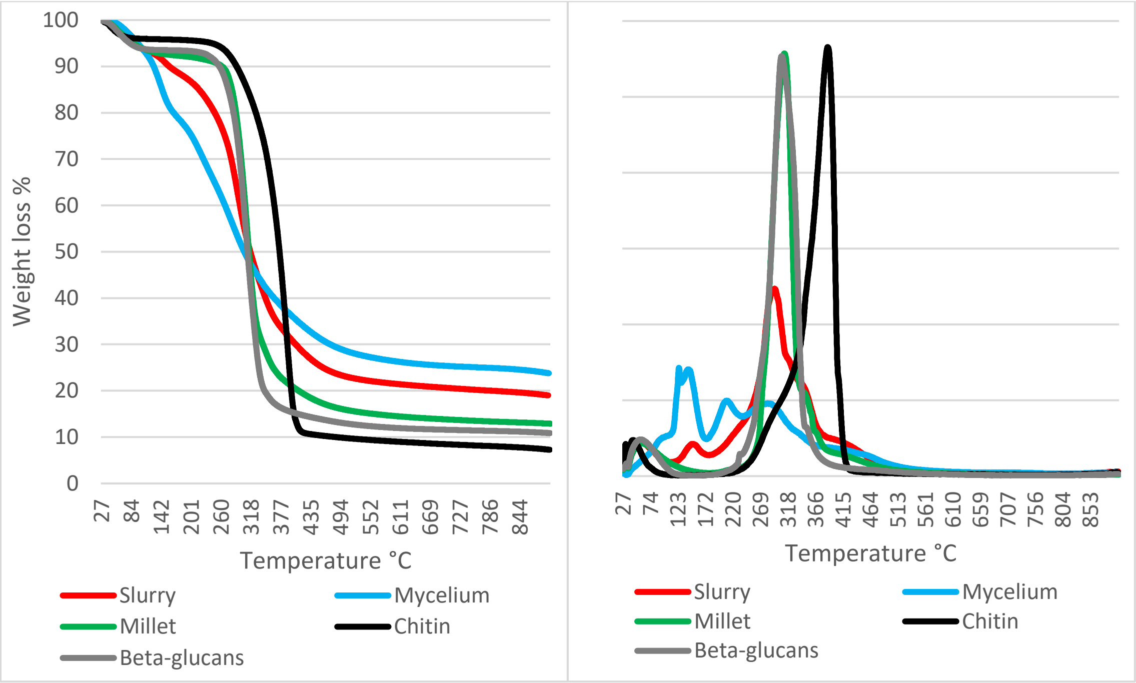

Figure S1: TGA (left) and DTGA (right) profiles of strain 1 (pure mycelium and slurry mat) compared to reference materials.

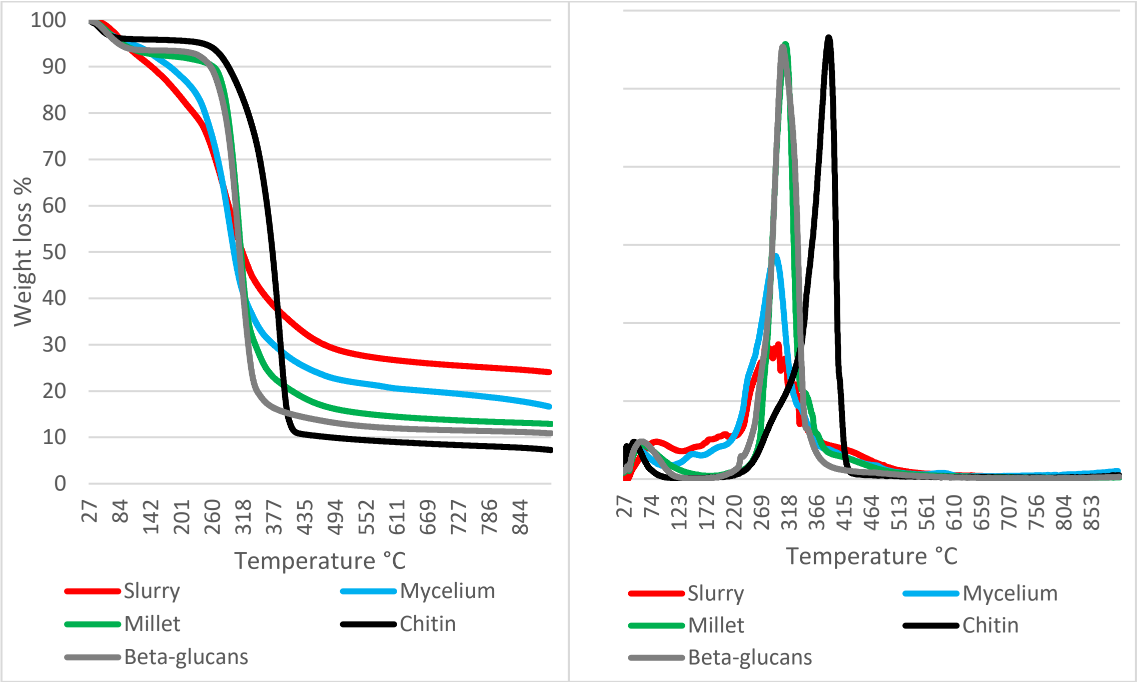

Figure S2: TGA (left) and DTGA (right) profiles of strain 2 (pure mycelium and slurry mat) compared to reference materials.

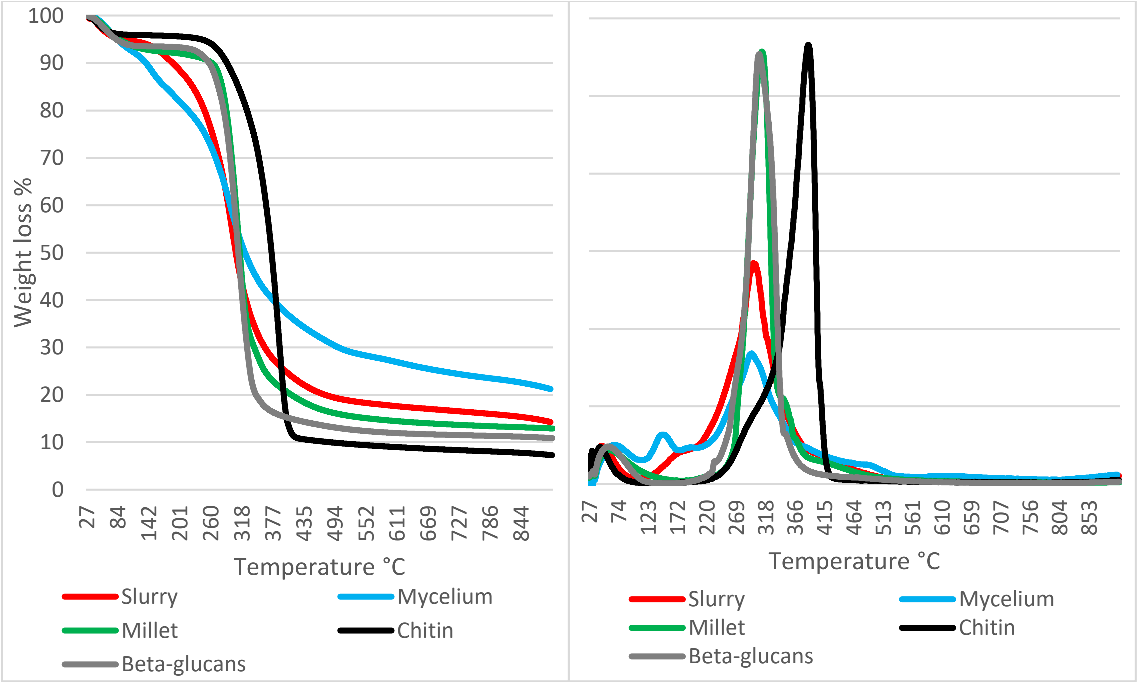

Figure S3: TGA (left) and DTGA (right) profiles of strain 3 (pure mycelium and slurry mat) compared to reference materials.

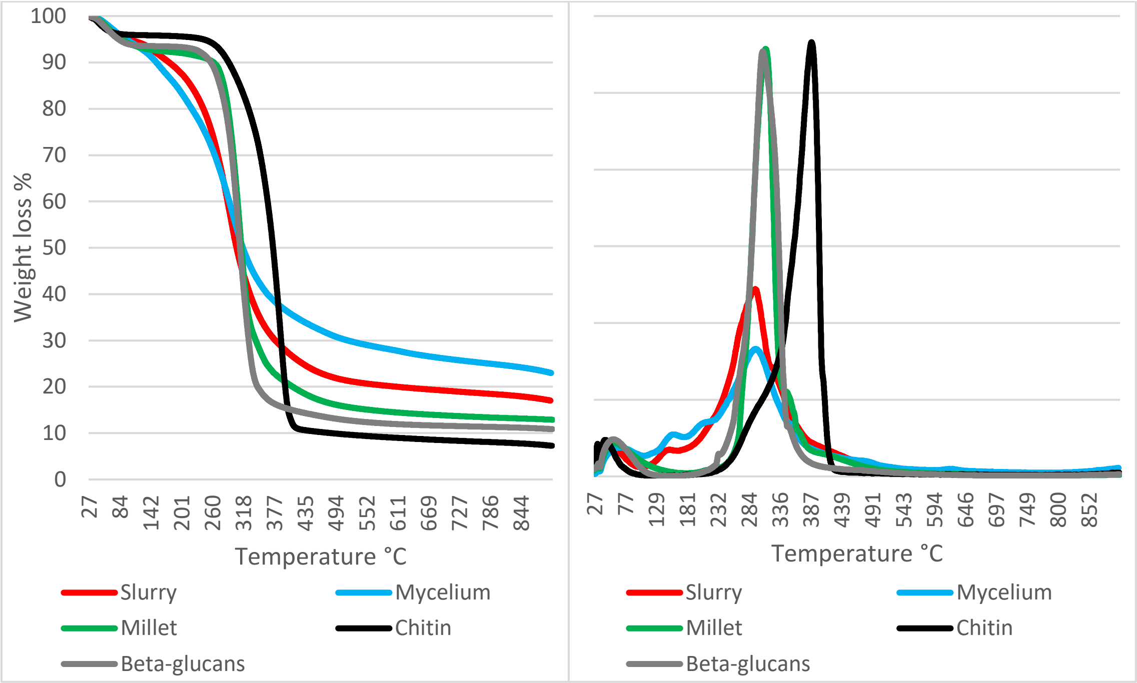

Figure S4: TGA (left) and DTGA (right) profiles of strain 4 (pure mycelium and slurry mat) compared to reference materials.

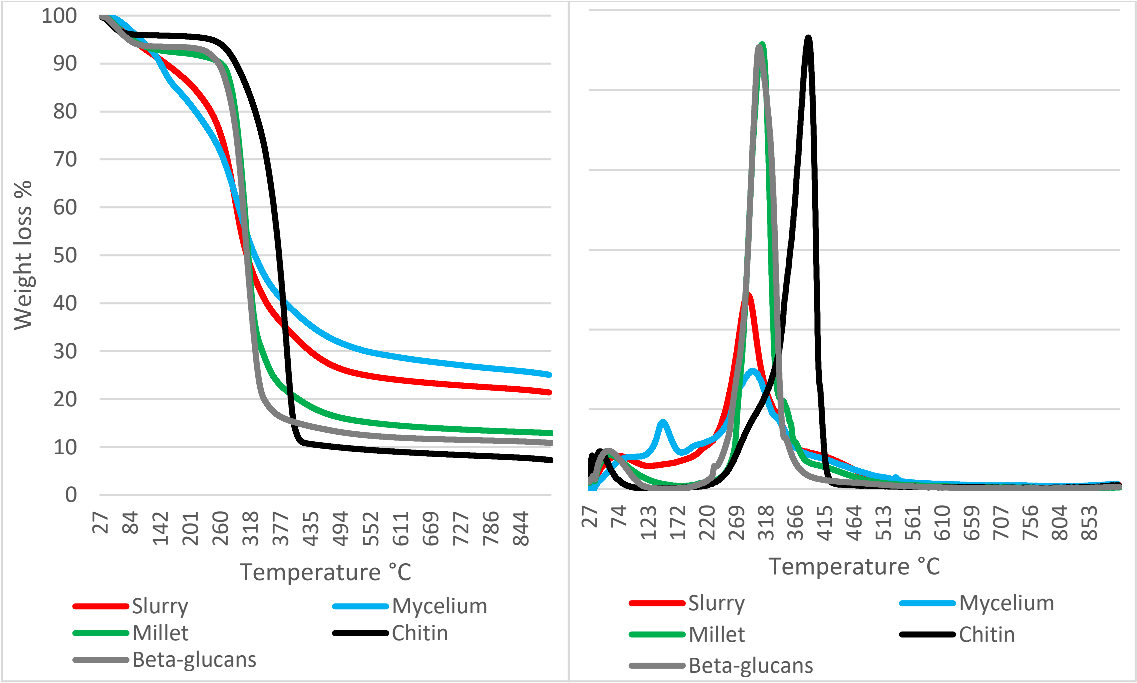

Figure S5: TGA (left) and DTGA (right) profiles of strain 5 (pure mycelium and slurry mat) compared to reference materials.

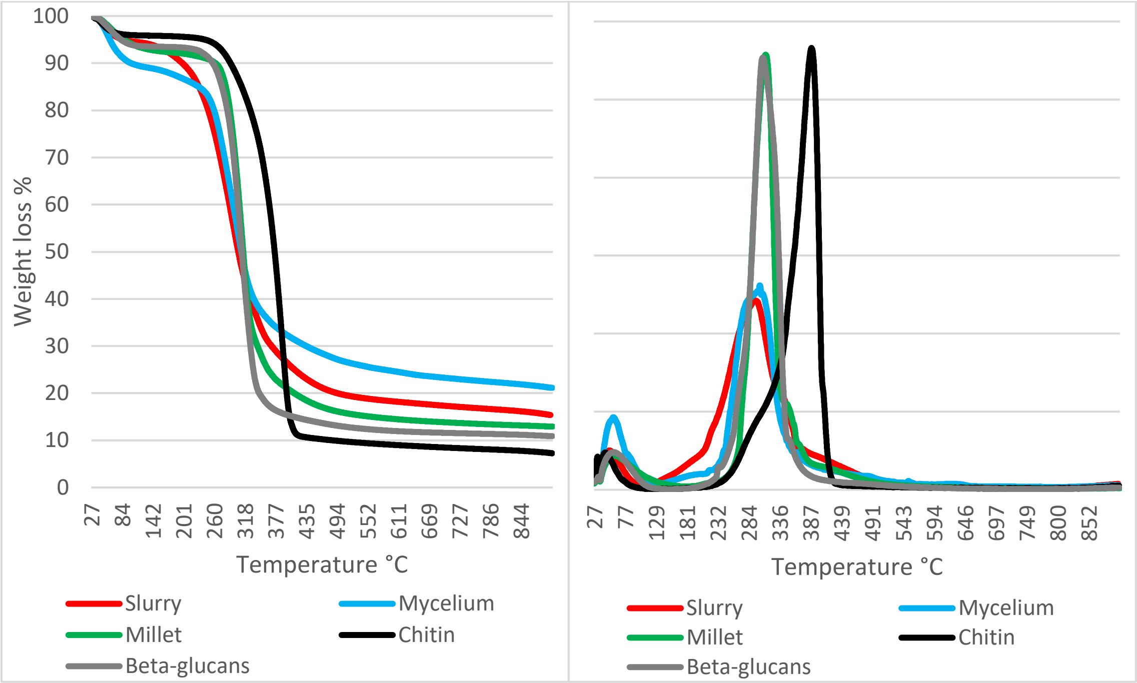

Figure S6: TGA (left) and DTGA (right) profiles of strain 6 (pure mycelium and slurry mat) compared to reference materials.

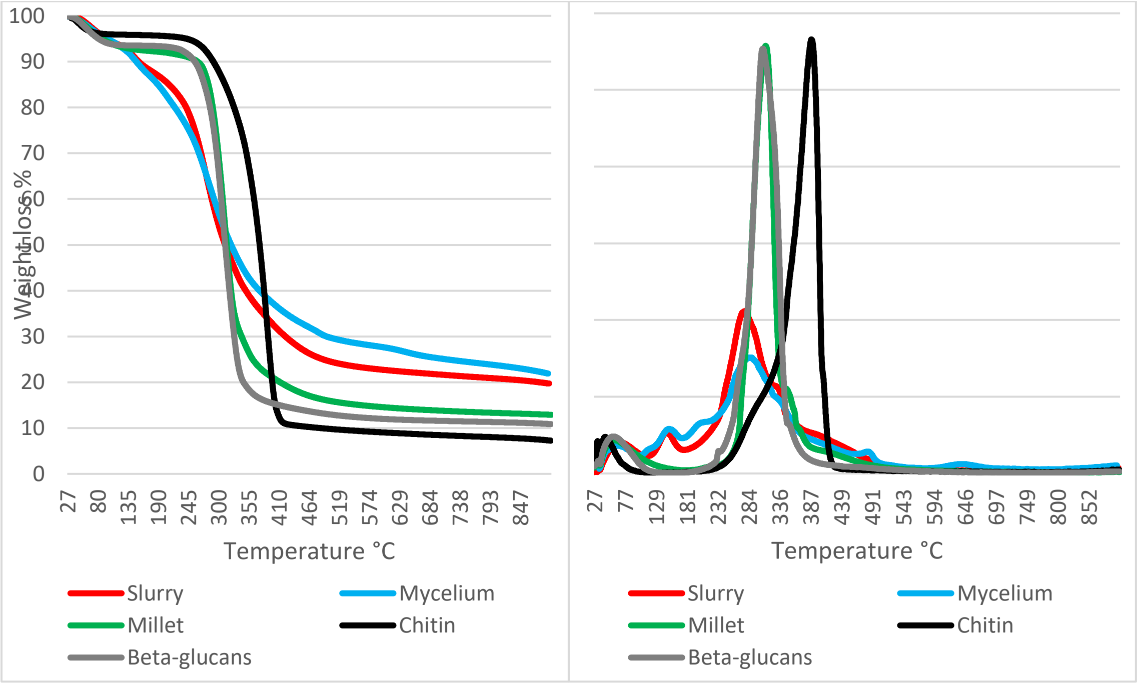

Figure S7: TGA (left) and DTGA (right) profiles of strain 7 (pure mycelium and slurry mat) compared to reference materials.

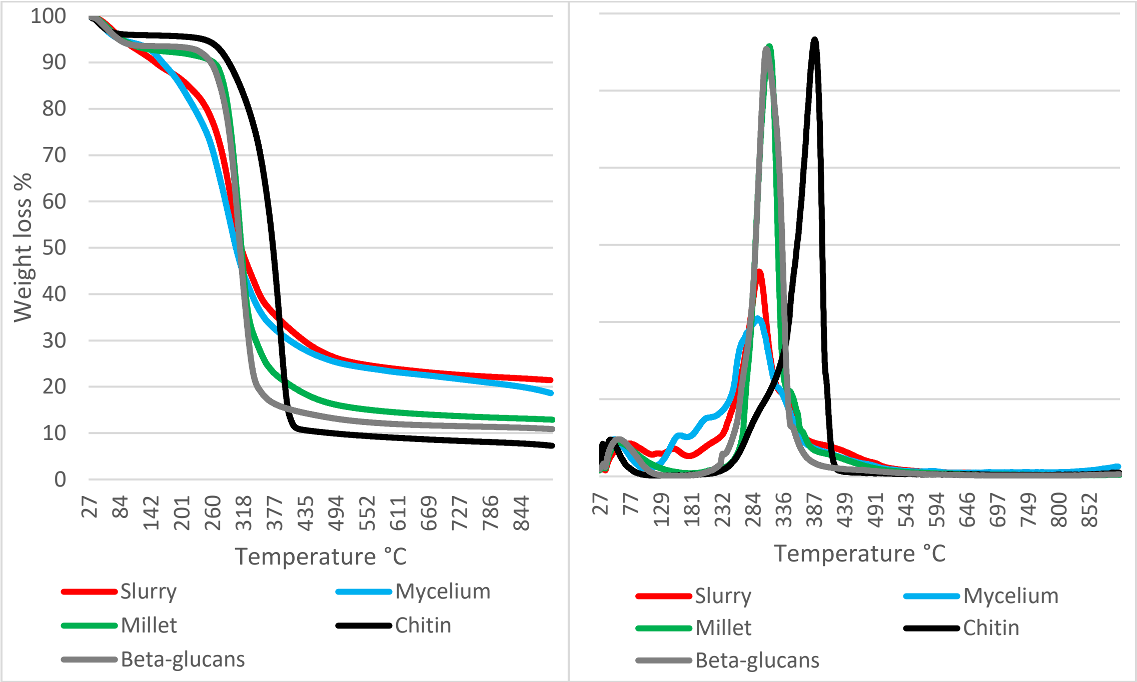

Figure S8: TGA (left) and DTGA (right) profiles of strain 8 (pure mycelium and slurry mat) compared to reference materials.

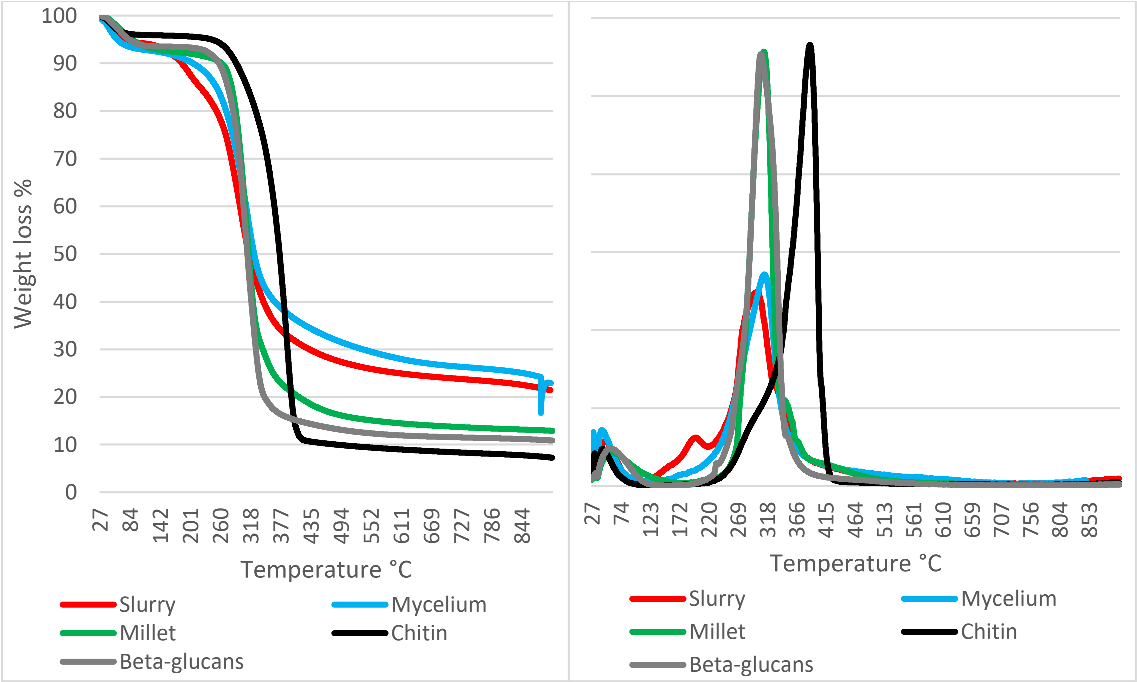

Figure S9: TGA (left) and DTGA (right) profiles of strain 9 (pure mycelium and slurry mat) compared to reference materials.

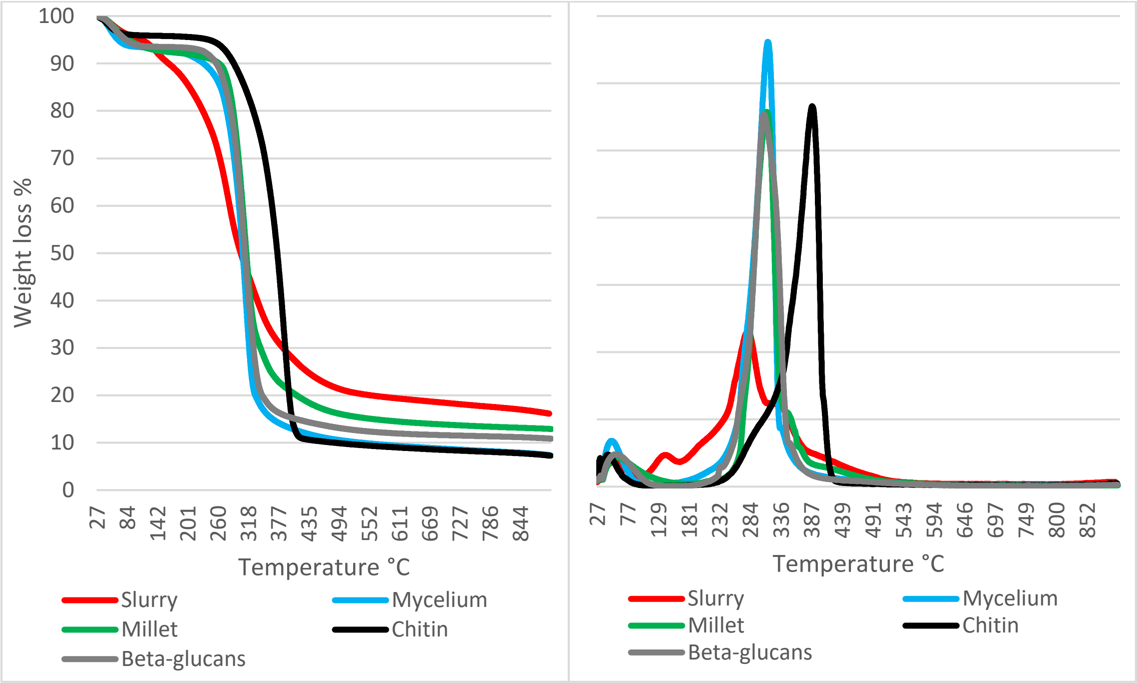

Figure S10: TGA (left) and DTGA (right) profiles of strain 10 (pure mycelium and slurry mat) compared to reference materials.

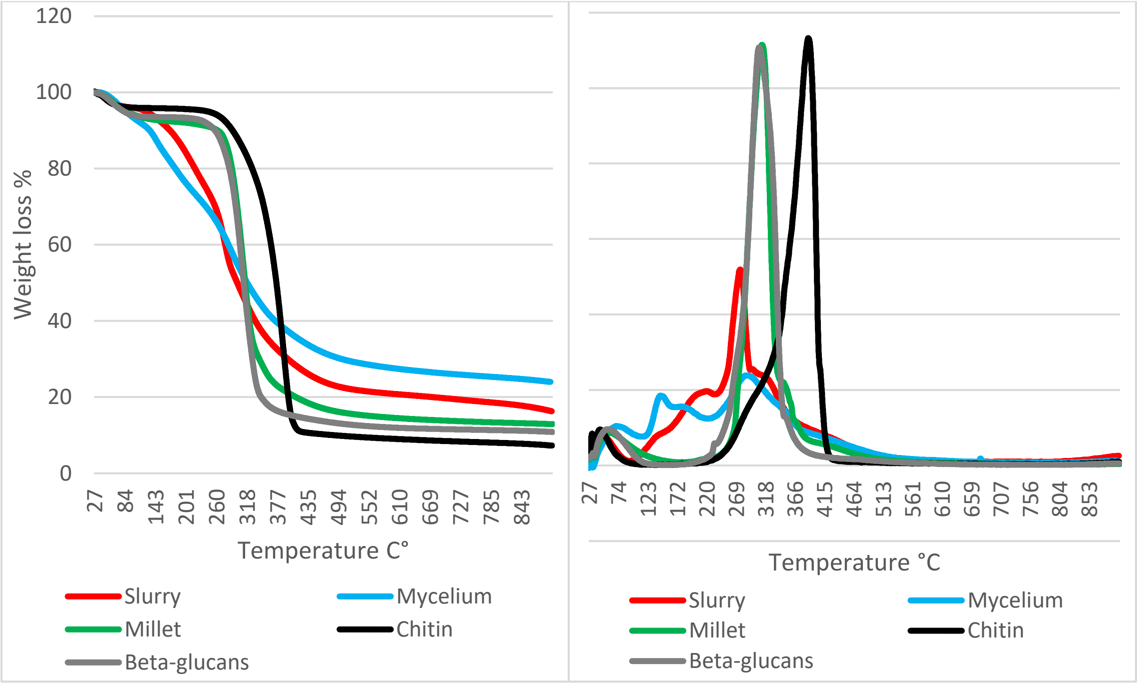

Figure S11: TGA (left) and DTGA (right) profiles of strain 11 (pure mycelium and slurry mat) compared to reference materials.

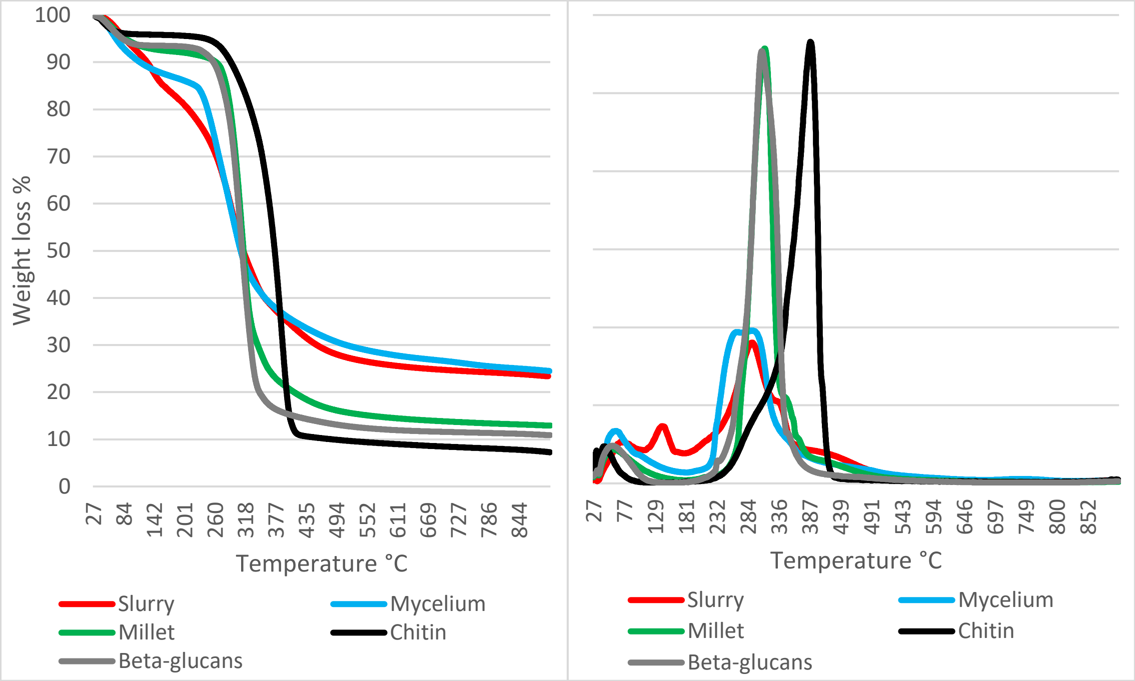

Figure S12: TGA (left) and DTGA (right) profiles of strain 12 (pure mycelium and slurry mat) compared to reference materials.

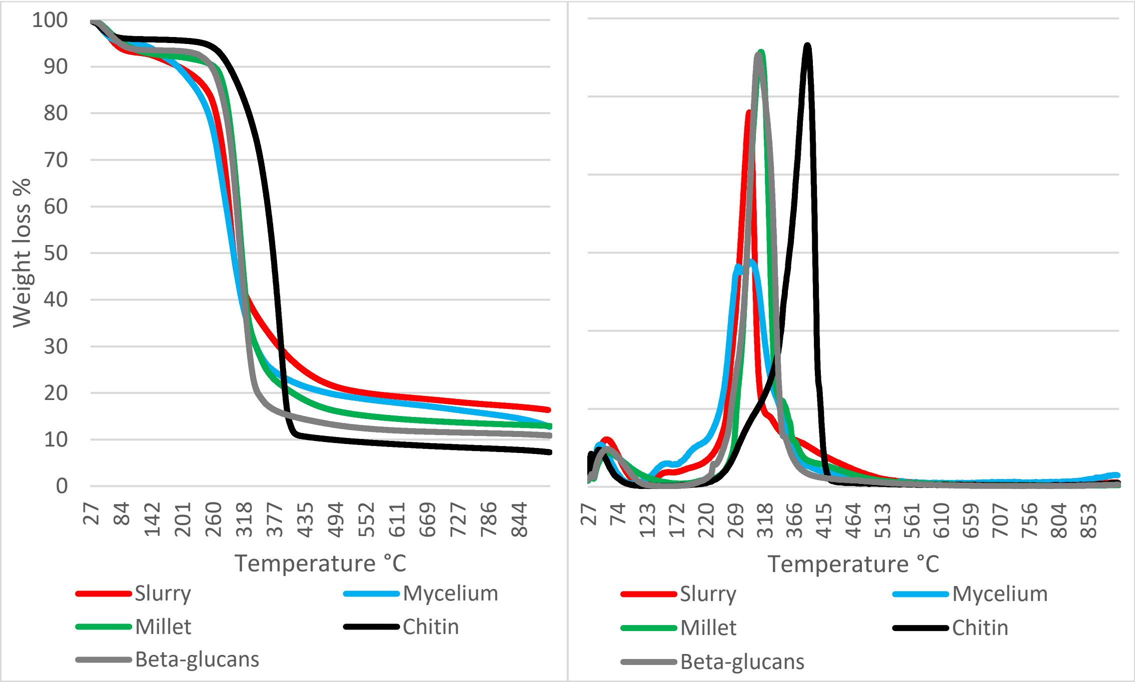

Figure S13: TGA (left) and DTGA (right) profiles of strain 13 (pure mycelium and slurry mat) compared to reference materials.

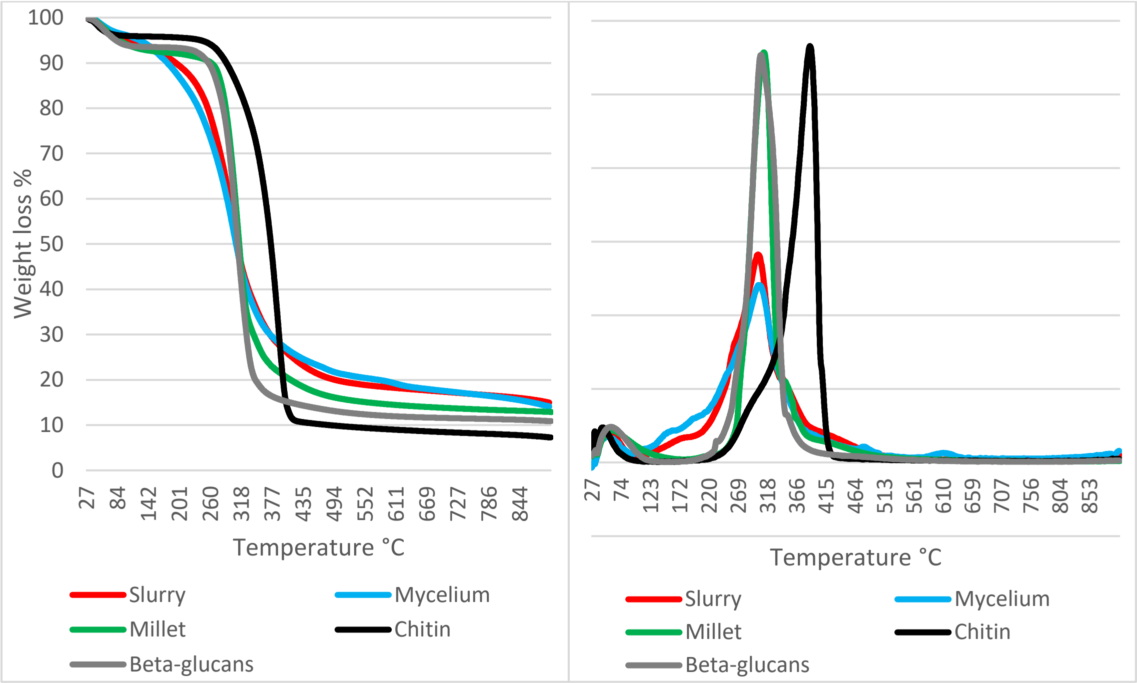

Figure S14: TGA (left) and DTGA (right) profiles of strain 14 (pure mycelium and slurry mat) compared to reference materials.

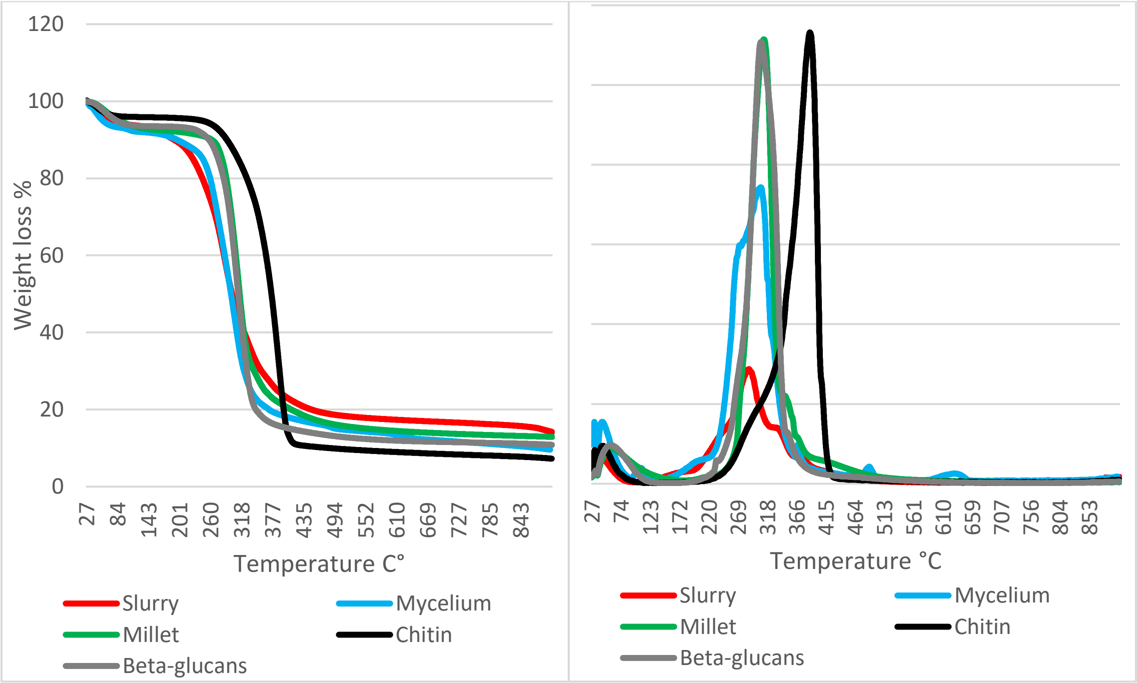

Figure S15: TGA (left) and DTGA (right) profiles of strain 15 (pure mycelium and slurry mat) compared to reference materials.

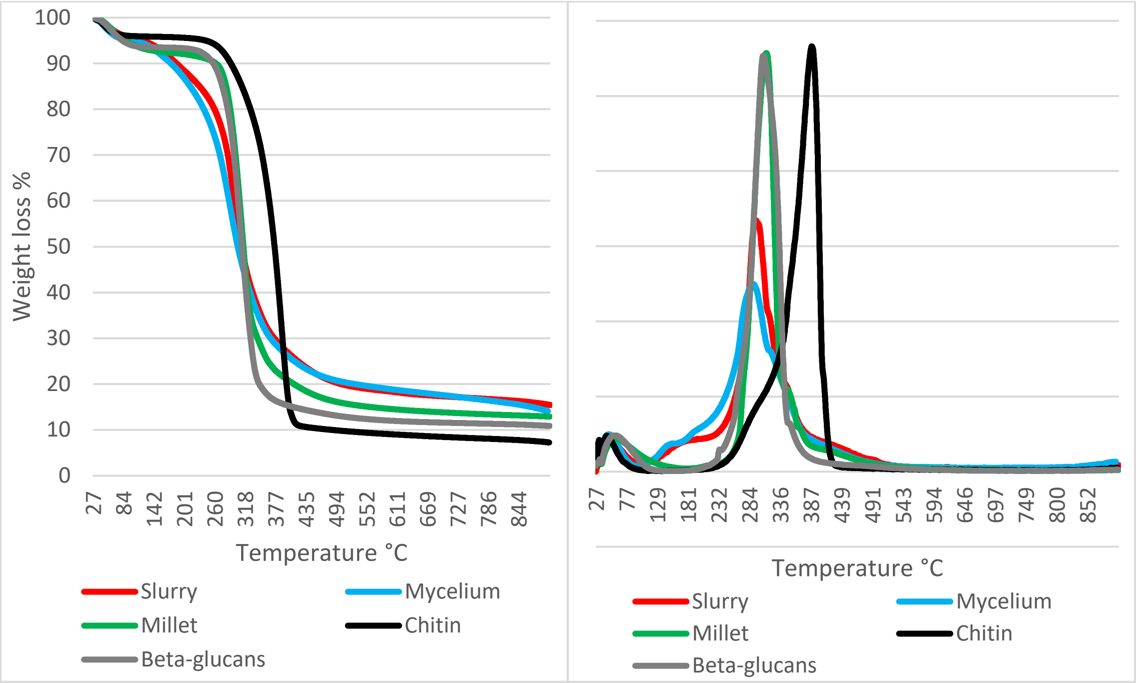

Figure S16: TGA (left) and DTGA (right) profiles of strain 16 (pure mycelium and slurry mat) compared to reference materials.

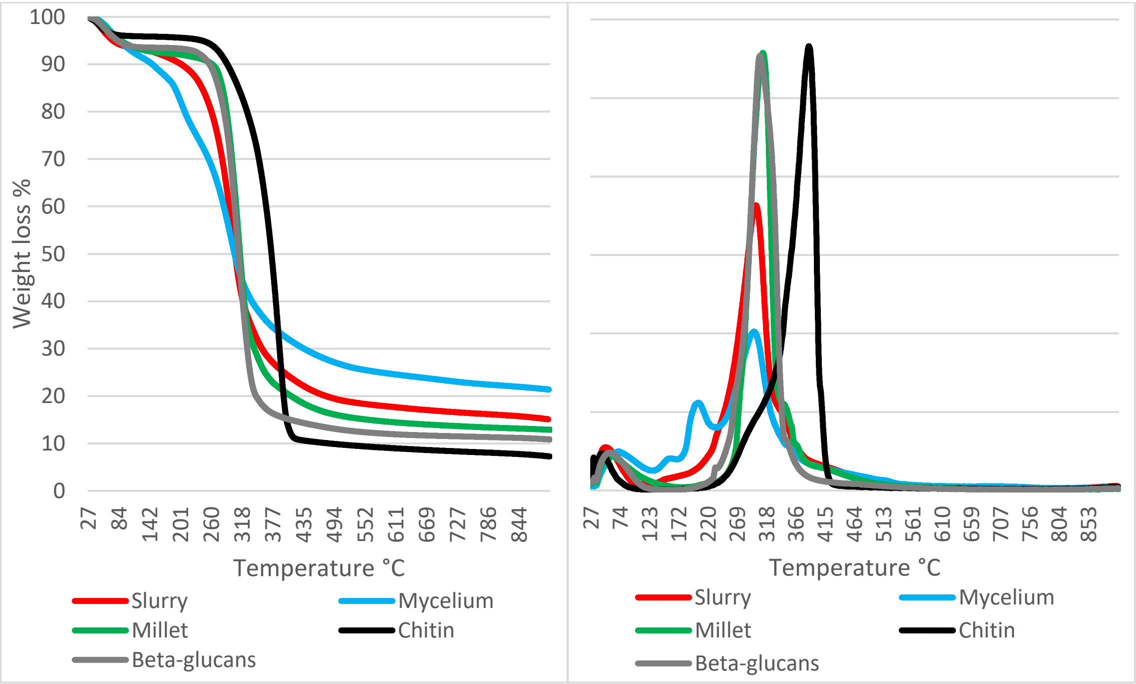

Figure S17: TGA (left) and DTGA (right) profiles of strain 17 (pure mycelium and slurry mat) compared to reference materials.

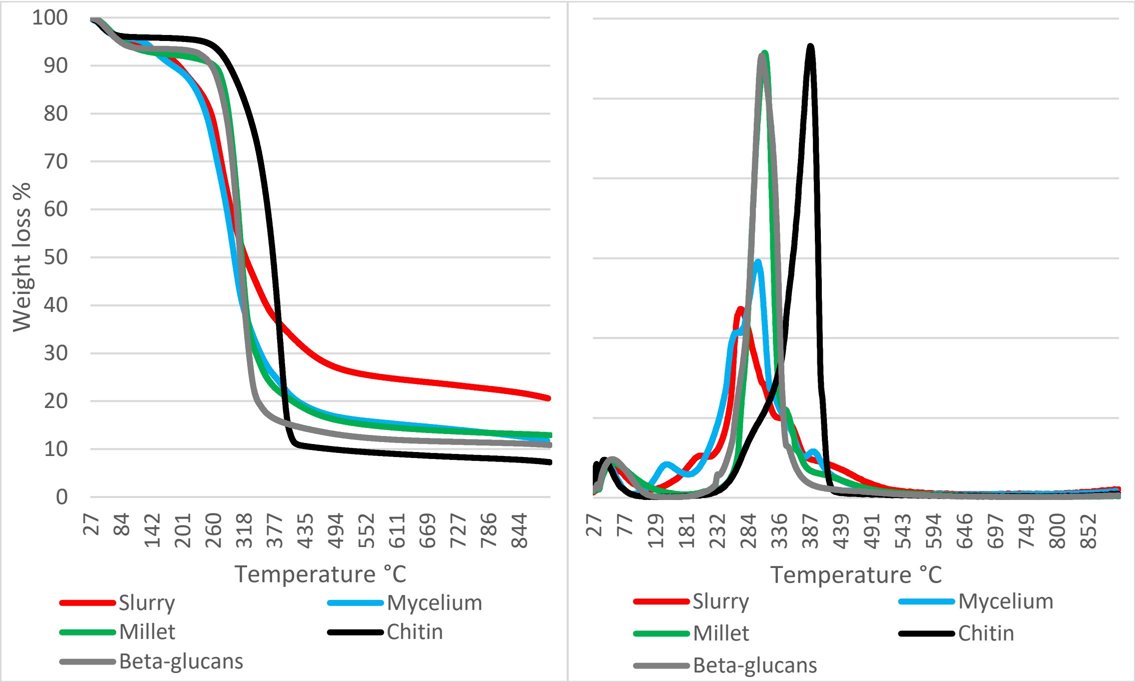

Figure S18: TGA (left) and DTGA (right) profiles of strain 18 (pure mycelium and slurry mat) compared to reference materials.

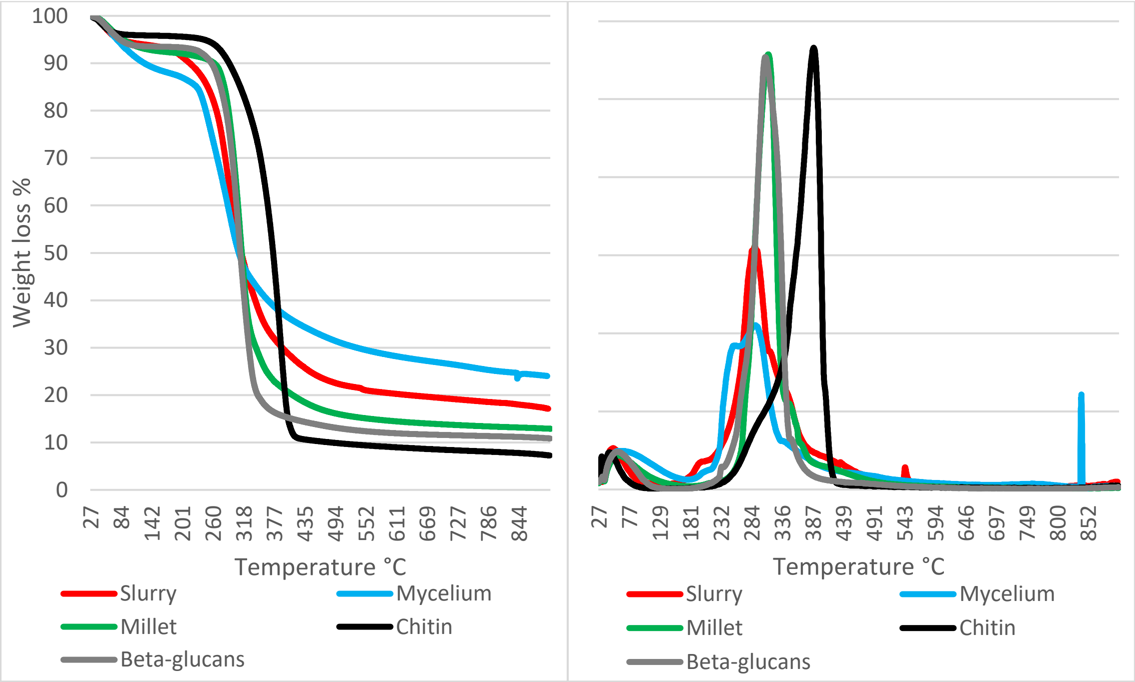

Figure S19: TGA (left) and DTGA (right) profiles of strain 19 (pure mycelium and slurry mat) compared to reference materials.

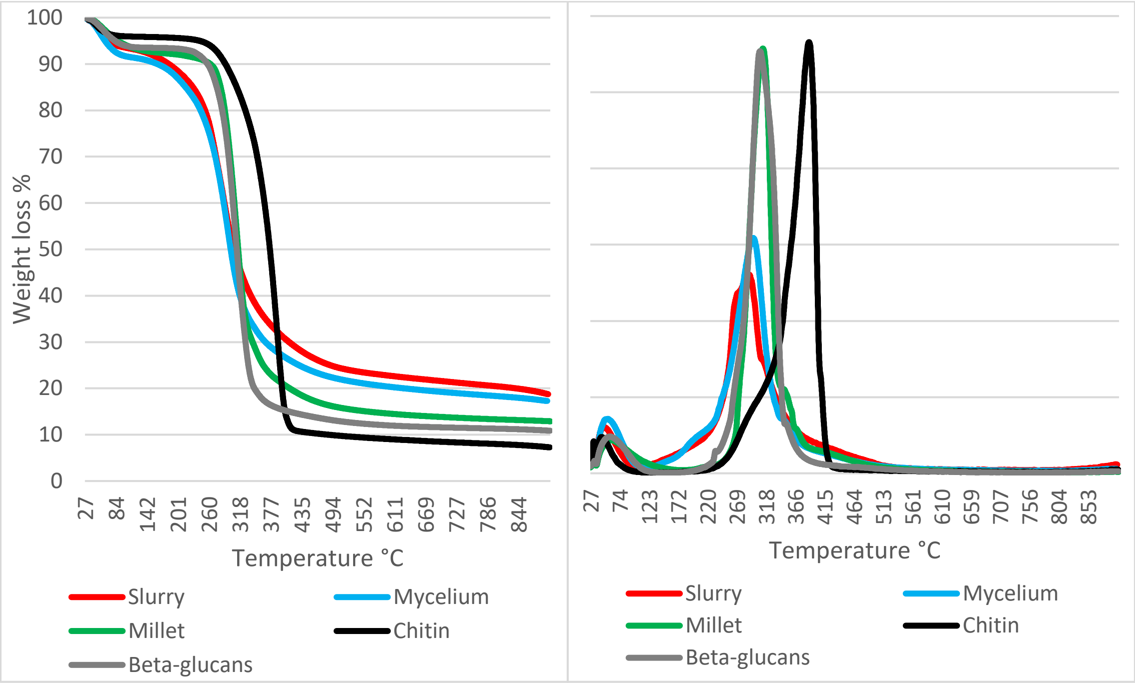

Figure S20: TGA (left) and DTGA (right) profiles of strain 20 (pure mycelium and slurry mat) compared to reference materials.

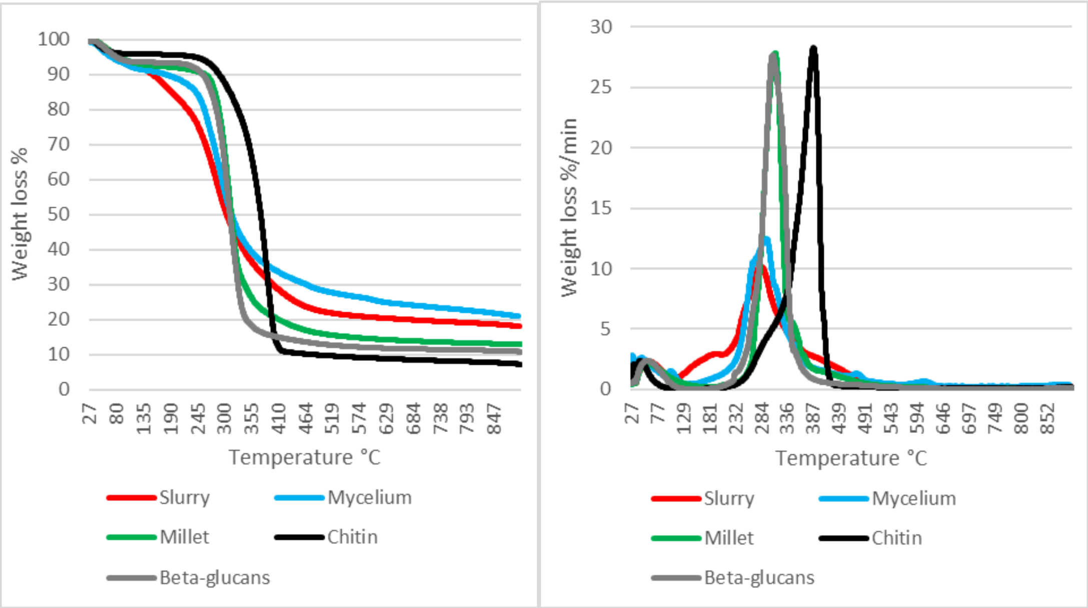

Figure S21: TGA (left) and DTGA (right) profiles of strain 21 (pure mycelium and slurry mat) compared to reference materials.

Table S1: ITS sequences of the strains and main parameters on Mycobank Molecular ID and NCBI.

strain code in the paper	species	MFSC code	ITS sequence	Score	Similarity
1	*Abortiporus biennis* (Bull.) Singer	064-18	TCTACCTGATTTGAGGTCAGATTTCAAGTATATAAGTTTGTCTGA GTAAACAGACGGTTAGAAGCATGAACTATAAATTTTGTTACAACA TCTACAGCGCAGATAATTATCACACTGAAAAATGTGCAACACATT CACACTAATACATTTCAGAGGAGCTGATGTTGATTAAAACACCGG CATACCTCCAAGTCCAATTCTGACAACAAAAGTTGAGAGAATTGA GAATACCATGACACTCAAACAGGCATGCTCCTCGGAATACCAAG GAGCGCAAGGTGCGTTCAAAGATTCGATGATTCACTGAATTCTG CAATTCACATTACTTATCGCATTTCGCTGCGTTCTTCATCGATGCG AGAGCCAAGAGATCCGTTGCTGAAAGTTGTATTATTTAATTGCGT TAAACGCGAGTATTACATTCTTTAAACTGAAGCGTATAAATGTGT AAACCACAGACTTTAAATCTTCAATTGACTTTCGTCAAACTCGCG GTGGCAAGCTCGAACGTCCATCAATAAGAAAATATAAAAATCCA TGTAAAGTGCACAGAGGTGTAAAGAATTTGGAAATAACCGAGGC GTGCACATGCCTAATAAAAGGCCAGCAACAACCAAGGCATTAAA AATTCAGTAATGATCCTTCCGCAGGTTCACCTACGGAAG	1030.23	99.85%
2	*Bjerkandera adusta* (Willd.) P. Karst.	101-19	TGCGGAAGGATCATTATCGAGTTTTGAACGGGTTGTCTGCTGGCT CGCAAGGGCATGTGCACGCCTGTCTCATCCACTCTAACTTCTGTG CACTTTTCATAGGCCGGCTTGTGGGTGCGTTCGCGCACTTGTAGG TGTCGGGCTTATGCTTTATTACAAACGATTCAGTTTTAGAATGTCA TACTTTGCTATAACGCAATTTATATACAACTTTCAGCAACGGATCT CTTGGCTCTCGCATCGATGAAGAACGCAGCGAAATGCGATAAGT AATGTGAATTGCAGAATTCAGTGAATCATCGAATCTTTGAACGCA CCTTGCGCTCCTTGGTATTCCGAGGAGCATGCCTGTTTGAGTCTC ATGGAATTCTCAACCTTCGGCTTCATTGACGAAGGCTTGGACTTG GAGGTCGTGCCGGCTCTCGTAGTCGGCTCCTCTGAAATGCATTAG TGCGAACGTTACCGACCGCTTCAGCGTGATAATTATCTGCGTTGC TGTGGAGGGTATTCTAGTGTTCACGCTTCTAACCGTCTTCGGACA AATTTCTGAACTCTGAGCTCAAATCAGTAGGACTACCCGCTGAAC TTAAGCATATCAT	905.01	99.67%
3	*Coriolopsis gallica* (Fr.) Ryvarden	086-19	GACGGGTTGTACTGGCTCTCCGGAGGCCTGTGCACCCCTGCTCAT CCACTCTACACCTGTGCACTACTGTGGGTATCGGAGGGCGTCCA GTCGTTTGCGGCGAGGCGTTAACCGTGCCTACGTCTTATTACAAA CGCTTCAGTATCAGAATGTGTATTGCGATGTAACGCATCTATATA CAACTTTCAGCAACGGATCTCTTGGCTCTCGCATCGATGAAGAAC GCAGCGAAATGCGATAAGTAATGTGAATTGCAGAATTCAGTGAA TCATCGAATCTTTGAACGCACCTTGCGCTCCTTGGTATTCCGAGG AGCATGCCTGTTTGAGTGTCATGAAATTCTCAAACCCATAAGTCT TTGCGGGCTTACGGGCTTTGGACTTGGAGGCTTTGTCGGCGACC GCGAGGTCTGTCGACTCCTCTCAAATGCATTAGCTTGATTCCTTG CGGATCGGCTCTCGGTGTGATAATTGTCTACGCCGTGACCGTGA AGCGTTTTGGCGAGCTTCTAATCGTCTCTTACGAGACAGTTTACA TTGACCTCTGACCTCAAATCAGGTAAGACTACCCGCTGAACTTAA CCATATCAATAAGCGGAGGAA	843.20	98.27%

4	*Coriolopsis gallica* (Fr.) Ryvarden	121-19	GACGGGTTGTAGCTGGCCTTTCCGGAGGCCTGTGCACGCTCTGC TCATCCACTCTACACCTGTGCACTTACTGTGGGTATCGGAAGGCG TCGAGTCGTTTGCGGCGAGGCGTTAACCGTGCCTACGTCTTATTA CAAACGCTTCAGTATCAGAATGTGTATTGCGATGTAACGCATCTA TATACAACTTTCAGCAACGGATCTCTTGGCTCTCGCATCGATGAA GCCCGCAGCGAAATGCGACAAGTAATGTGAATTGCAGAATTCAG TGAATCATCGAATCTTTGAACGCACCTTGCGCTCCTTGGTATTCCG AGGAGCATGCCTGTTTGAGTGTCATGAAATTCTCAAACCCATAAG TCTCCGCGGGCTTACGGGCTTTGGACTTGGAGGCTTTGTCGGCG ACCGCGAGGTCACGTCGACTCCTCTCAAATGCATTAGCTTGATTC CTTGCGGATCGGCTCTCGGTGTGATAATTGTCTACGCCGTGACCG TGAAGCGTTTTGGCGAGCTTCTAATCGTCTCTTACGAGACAGTTT ACATTGACCTCTGACCTCAAACGGAGGCA	827.35	97.85%
5	*Coriolopsis trogii* (Berk.) Domanski	027-18	GCCTTCCAAGTTCCAAAGCCCCTAAGCCCGCAAAGACTTATGTTT AGAATTTTCATGACACTCAAACCGGCATGCTCCTGGAAATACCCA AGAGCGCAAGGGTTTCAAAGATTGGATGATTCCACTGAATTCTG CAATTCACATTACTTATCGCATTTCGTGGTTTCTTCATTGATGGAG AGCCAAGAGATTCCGTGCGAAAGTTGTATATAGATGCGTTACAT CGCAATACACATTTTGATAGTGAATCGTTTGTAGTAAAACGTAGG CACGTTAACGCCTCGCCGTAAACGACGCGACACCTCCCGATACCC ACAGTAAGTGCACAGGTGTAGAGTGATGAGCAGGCGTGCACAT GCCTCCGGAGAAGCCAGCTACAACCCATTTCAAAACTCGATAATG ATCCTTCTTCCGTAGGTGAACCTGCGGAAGGATCATTATCGAGTT TTGAAATGGGTTGTAGCTGGCTTCTCCGGAGGCATGTGCACGCC CTGCTCATCCACTCTACACCTGTGCACTTACTGTGGGTATCGGGA GGTGTCGCGTCGTTTACGGCGAGGCGTTAACCGTGCCTACGTTTT ACTACAAACGATTCAGTATCAGAATGTGTATTGCGATGTAACGCA TCTATATACAACTTTCAGCAACGGATCTCTTGGCTCTCGCATCGAT GAAGAACGCAGCGAAATGCGATAAGTAATGTGAATTGCAGAATT CAGTGAATCATCGAATCTTTGAACGCACCTTGCGCTCCTTGGTAT TCCGAGGAGCATGCCTGTTTGAGTGTCATGAAATTCTCAAACCCA TAAGTCTTTGCGGGCTTACGGGCTTTGGACTTGGAGGCTTGTCG GCGACCGTGAGGTCACATCGACTCCTCTCAAATGCATTAGCTTGA TTCCTTGCGGATCGGCTCTCGGTGTGATAATTGTCTACGCCGTGA CCGTGAAGCGTTTTGGCAAGCTTCTAACCGTCTCTAACGAGACAG CTTACTTTGACCTCTGACCTCAAATCAGGTAGACTCCCCCAGAACT TATGCATATGGTTAACCGGCGGAAAGGAACATTATCGAGTTTTG AAATGGGTTGTAGCTGGCTTCTCCGGAGGCATGTGCACGCCTGC TCATCCACTCTACACCTGTGCACTTACTGTGGGTATCGGGGAGGT GTCGCGTCGTTTAGGCGAGGCGTTAACGTGCCTACGTTTTACTAC AAACGATTCATTATCAAAATGTGTATTGCGATGTAACGCTCTATA TACACTTTAGCAACGGATCTCTTGGCTCTCCATCGATGAAGAAAC GCACCGAAATGCGATAAGTTAATGTGGAATTGCAGAAATTCATT GAAATCATCAAATCTTTGAACCCCTTGCCCTCTTGGGTATTCCAG GGAGCATGCCGTT	965.24	95.33%

6	*Daedaleopsis confragosa* (Bolton) J. Schröt.	155-19	CTGCGGAAGGATCATTAACGAGTTTTGAGGGGTTGTAGCTGGCC<br>TTCCGAGGCATGTGCACGCCCTGCTCATTCCACTCTACACCTGTG<br>CACTTACTGTGGGTCTCAGGCGAGCGTCGGTCGCTTCGCGGCGT<br>CGTCGTTCAACTGGGCTCACGTTTTACTACAAACTATTAAAGTATC<br>AGAATGTCTACTGCGAATTAACGCATTTAAATACAACTTTCAGCA<br>ACGGATCTCTTGGCTCTCGCATCGATGAAGAACGCAGCGAAATG<br>CGATAAGTAATGTGAATTGCAGAATTCAGTGAATCATCGAATCTT<br>TGAACGCACCTTGCGCTCCTTGGTATTCCGAGGAGCATGCCTGTT<br>TGAGTGTCATGAAATTCTCAACCTAACGAGTCTTTGCGGGCTCGG<br>TAGGCTTGGACTTGGAGGTTCTTGTCGGCCTAACGGTCGGCTCCT<br>CTTAAATGCATTAGCTCGGTTCCTTGCGGATCGGCTCACGGTGTG<br>ATAATTGTCTACGCCGCGACCGTTGAAGCGTTTTGGCCGGCTTCT<br>AATCGTCTCGTTGGAGACACTACTCTTTATGACCTTCTGACCTCAA<br>ATCAGGTAGGACACCGCTGAACTTAAGCATATCAATAAGCCGGA<br>AGGAGATCATTAACGAGTTTTGAAAGGGGTTGTAGCTGGCCTTC<br>CGAGGCATGTGCAGCCTGCTCATTCACTCTACACCTGTGCACTTA<br>CGTGGGTCTCAGGCGAGCGTCGGTCGCTTCCGGCGTCGTCGTTC<br>ACTGGGCTCCGTTTTACTACAAACTATTAAAGTATCAGAATGTCT<br>ACGCGAATTAAGCACTTAAATAAACTTTCGCAACGGATCTCTTGG<br>CTCTGCATCGATTAAGAAGCACGAAATGCGATAAGTAATGTGAA<br>TTGCACAATTCGTGGAATCTCGAAACTTTGAACACCTTGCGCTCTT<br>GGGTATTCCAGGGAGCAGCTGTTTTGAGTGTCAGGAAAATTCAA<br>CTAACAGGTCTTTGCGGGCTCGGTAGGCTTGGGACTTGGAAGGT<br>TCTTGTTCGCCTAACGGTGGCTCTCTTAAATGCATTAACTCGGTTC<br>CTTGCGGAATGGCCCCGGGTTTGGATAATTGTTCTCCCCCCGCCT<br>TTAAACCCTTTTTGGC	920.86	92.54%
7	*Daedaleopsis confragosa var. tricolor* (Bull.) Bondartsev & Singer	148-19	GCGGAAGGATCATTAACGAGTTTTGAGGGGTTGTAGCTGGCCTT<br>CCGAGGCATGTGCACGCCCTGCTCATTCCACTCTACACCTGTGCA<br>CTTACTGTGGGTCTCAGGCGAGCGTCGGTCGCTTCGCGGCGTCG<br>TCGTTCAACTGGGCTCACGTTTTACTACAAACTATTAAAGTATCA<br>GAATGTCTACTGCGAATTAACGCATTTAAATACAACTTTCAGCAA<br>CGGATCTCTTGGCTCTCGCATCGATGAAGAACGCAGCGAAATGC<br>GATAAGTAATGTGAATTGCAGAATTCAGTGAATCATCGAATCTTT<br>GAACGCACCTTGCGCTCCTTGGTATTCCGAGGAGCATGCCTGTTT<br>GAGTGTCATGAAATTCTCAACCTAACGAGTCTTTGCGGGCTCGGT<br>AGGCTTGGACTTGGAGGTTCTTGTCGGCCTAACGGTCGGCTCCTC<br>TTAAATGCATTAGCTCGGTTCCTTGCGGATCGGCTCACGGTGTGA<br>TAATTGTCTACGCCGCGACCGTTGAAGCGTTTTGGCCGGCTTCTA<br>ATCGTCTCGTTGGAGACACTACTCTTTATGACCTTCTGACCTCAAA<br>TCAGGTAGGACTACCCGCTGAACTTAAGCAT	935.13	100%
8	*Fomes fomentarius* (L.) Fr.	179-19	GCGGAAGGATCATTAACGAGTTTGCTGGGTTGTAGCTGGCCTTC<br>CGAGGCATGTGCACGCCCTGCTCATCCACTCTACACCTGTGCACT<br>TACTGTGGGATTTCAGGTGCGTCGCTTTGCGGCGGCGTCACTCG<br>GCCCACGTTTTCTTTACAAACTATCGAAGTAACAGAATGTTTATTG<br>ATGTAACGCATCTATAATACAACTTTCAGCAACGGATCTCTTGGC<br>TCTCGCATCGATGAAGAACGCAGCGAAATGCGATAAGTAATGTG<br>AATTGCAGAATTCAGTGAATCATCGAATCTTTGAACGCACCTTGC<br>GCTCCTTGGTATTCCGAGGAGCATGCCTGTTTGAGTGTCATGAAA<br>TTCTCAACCTATAAACTTTTGTGGGTTTGTAGGGTTGGCTATTGG<br>AGGCTTTTGCTGGCCCTCGTTTGAGTCAGCTCCTCTCAAATGCATT<br>AGCTTGGTTCCTTGCGGATCGGCTGTCGGTGTGATAATGTCTACG<br>CCGCGACCGTGAAGCGTTTGGAGAGCTTCTAATGGTCTCGTCAG<br>AGACAGCTTTTATGAACTCTGACCTCAAATCAGGTAGACACCCGC<br>TGAACTTAAGCATAT	866.97	99.64%

9	*Fomitiporia mediterranea* M. Fisch.	079-18	TTTTTTGTTTTGGGAGGGCCCCAACGTCTCCCGTGTGATAGAGGT<br>GAAATTATTGTTATTTTCTCCCACGGAGGCCCTTTACACTTTTCCC<br>GAGACCCAGCAAAGTGTGTGTTATATATGTTAAAGTTCCAAGGG<br>TGTTCCCCGGTATTTGTGTGGTGCCCCCGGTTTTTTTTTTTATTTCT<br>TATTTGCGGGAGAGAGGTTCCCCCCGCTTTCTCCCGTTGGATTGC<br>CTATGGGCCGGCCCCCACTATACTATTACCAACTTTGATAATGTG<br>CACCGGGGGTGTGGTTGATGAGTGTGAAGGCGAGCACATGTCC<br>CCATATTTGCACCAGCCTCCAACTTCCACTTTCCAATTCGTTTTTGT<br>TCCCTTCCTTTCCGTAGGGGTGACCTGCGGAAGGATCATTAACGA<br>GTTGGAACGTGGAGGTTGATGCTGGTGCATATATAGTGTACATG<br>TGCTCGCCTTCACACTCTTCATCCACTCAACCCCTGTGCACTTTATC<br>AGAGTTAGTAATAGTATTGTGGTGGCAGCCGTTTGTTATTCATTG<br>TTAGAAGCGGGGTAACTCTTTCTAGCAGTAGTAATAATAACAATC<br>TTGGTTCTACTACTATTACTGTGAACACTTTGACTTTTACTTATACA<br>AACACTTTGCTTGTTCTTGTGAATGTGTAATGCTCCTTGTGAGCG<br>AAATACAAATATACAACTTTCAACAACGGATCTCTTGGCTCTCGC<br>ATCGATGAAGAACGCAGCGAAATGCGATAAGTAATGTGAATTGC<br>AGAATTCAGTGAATCATCGAATCTTTGAACGCACCTTGCGCCCCT<br>TGGTATTCCGAGGGGCATGCCTGTTTGAGTGTCATGTAATTCTCA<br>ATCCTCTTTTTTCTTAATTGAAGGAGGGGGCTTGGACTTGGAGGT<br>TAATATATACATGCTGGTACTGTCTGTATCGGCTCCTCTAAAATGC<br>ATTAGCTGGACTGTAGTTCGCATTGTTTGGTGTAGTAATAGTTTT<br>CTATCTATATTCACTACAGTGCTTGCTTAGACTGTCTGCTTCTAAT<br>AGTCCGCCTATATGTCGGACAGGTACTCTGTTACCTTAAACCATTT<br>GACTCCTTTGACCTCAAATCAGGAGGACTACCCGCTGAACTTAAG<br>CATATCAATAAGGCCGGAGGAAGGATCATTAACCGAGTTGGAAC<br>GTGGAGGTTGATGCTGGTGCATATATAGTGTACATGTGCTCGCCT<br>TCACACTCTTCATCCACAACCCCTGTGCACTTTATCAGAGTTAGTA<br>ATAGTATTGTGGTGGCAGCCGTTTGTTATTCATTGTTTAGAAGCG<br>GGGGTAACTCTTTTCTAGCAGTAGTAAATATAAAACAATCTTGGT<br>TCTACTAACAATAACGTGGAAACCCTTGAACTTTTACTTTACACAA<br>ACCTTTGCTTGTTCTTGGAAAAGGGAAAAGGCCCCCGGGGAGGC<br>AAGAATACAAAATATCAACTTTTTAACACGGGGACTTCTGGGGCT<br>TCCCACAGAAAAAGAAA	1161.78	93.64%
10	*Fomitopsis iberica* Melo & Ryvarden	104-19	CTCTCCGCTTATTGATATGCTTAAGTTCAGCGGGTAATCTACCTGA<br>TTTGAGGTCAAGGTCAAAGTCATTGTCCAGTAAAGGACGATTGG<br>AAGCCGAGCCCATTGATATGCTTCACTGCAACGGCGTAGACAATT<br>ATCACACCGATAGCTGATCCGCAAAGGTTCGAGCTAATGCATTCA<br>AGAGGAGCCGATCACAAGTACCAGCAATAAACCTCCAAATCCAA<br>GCTCTATTCACAAAAGCAAATAGAGTTGAGAATTCCATGACACTC<br>AAACAGGCATGCTCCTCGGAATACCAAGGAGCGCAAGGTGCGTT<br>CAAAGATTCGATGATTCACTGAATTCTGCAATTCACATTACTTATC<br>GCATTTCGCTGCGTTCTTCATCGATGCGAGAGCCAAGAGATCCGT<br>TGCTGAAAGTTGTATTTAAATGCGTTAGACGCAAGAGTACATTCT<br>TTAAACTGAAGTAGTTTGTGATGATACATAGGAGGGCCTCCAAC<br>CAGAGCAAAGTCAATGAAGACCGTAGCTCCGATCACAAGCCAAC<br>CTACAGTGTGTGCACAGGTGTGTGAGATGGATAGTGATCAGGGC<br>GTGCACATGCCGCCCCAAAGAGACGGCCAGCTACAACCCCTTTC<br>AAAATTCATTAATGATCCTTCCGCAGGTTCACCTACGGAAG	930.37	99.83%

11	*Fomitopsis pinicola* (Sw.) P. Karst.	117-19	GTCAAAGTTATTGTCCCGTGAAGGACGATTGGAAGCCGAATAAT<br>ATGCTTCACAGTAACGGCGTAGACATTTTATCACACCGATAAGCT<br>GATCCACAAAAGGTTCGAGCTAATGCATTCAAGAGGAGCCGATC<br>ACAGGTACCGGCAATAAACCTCCAAGTCCAAGCTCTATTCACAAA<br>AGCAAATAGAGTTGAGAATTCCATGACACTCAAACAGGCATGCT<br>CCTCGGAATACCAAGGAGCGCAAGGTGCGTTCAAAGATTCGATG<br>ATTCACTGAATTCTGCAATTCACATTACTTATCGCATTTCGCTGCG<br>TTCTTCATCGATGCGAGAGCCAAGAGATCCGTTGCTGAAAGTTGT<br>ATTTAAATGCGTTAGACGCAAGAGGACATTCTTTTAACTGAAGTA<br>GTGTGTGATAAAACATAGGAAGGCCCCAACTAAAAAGCAGAGTC<br>GATGAAGACCCCACTCCAATCATAAGCCGACCTACAGTATGTGCA<br>CAGGTGTGTGAGATGGATAATGATCAGGGCGTGCACATGCCGCT<br>CAAACGAAACAGCCAGCTACAACCCCTTTCAGAATTCATTAATGA<br>TCCTTCCGCAGGTTCACCTACGG	946.22	99.83%
12	*Ganoderma carnosum* Pat.	161-19	CCCTAGGTGAACCTGCGGAAGGATCATTATCGAGTTCTGACTGG<br>GTTGTAGCTGGCCTTCCGAGGCACGTGCACGCCCTGCTCATCCAC<br>TCTACACCTGTGCACTTACTGTGGGTTTCAGATCTGTGAAGCGTG<br>CTCCTTGCGGGGCTTCGTGAAGCGCGTCTGTGCCTGCGTTTATCA<br>CAAACTCTATAAAGTATTAGAATGTGTATTGCGATGTAACGCATC<br>TATATACAACTTTCAGCAACGGATCTCTTGGCTCTCGCATCGATG<br>AAGAACGCAGCGAAATGCGATAAGTAATGTGAATTGCAGAATTC<br>AGTGAATCATCGAATCTTTGAACGCACCTTGCGCTCCTTGGTATT<br>CCGAGGAGCATGCCTGTTTGAGTGTCATGAAATCTTCAACCTACA<br>AGCCTTTGCGGTTTGTAGGCTTGGACTTGGAGGCTTGTCGGCCCT<br>TTGTCGGTCGGCTCCTCTTAAATGCATTAGCTTGATTCCTTGCGGA<br>TCGGCTCTCGGTGTGATAATGTCTACGCCGCGACCGTGAAGCGTT<br>TGGCGAGCTTCTAACCGTCTTCGCTTGAAGACAGCTTTATGACCT<br>CTGACCTCAAATCAGGTAGACTACCCGCTGAACTTAAGCATATCA<br>ATAAGCGGAG	979.51	99.69%
13	*Ganoderma lucidum* (Curtis) P. Karst.	137-19	GACCTGCGGAAGGATCATTTTGAGTTCTGACTGGGTTGTAGCTG<br>GCCTTCCGAGGCACGTGCACGCCCTGCTCATCCACTCTACACCTG<br>TGCACTTACTGTGGGTTTCAGATCTGTGAAGCGTGCCCCTTGCGG<br>GGCTTCGTGAAGCGCGTCTGTGCCTGCGTTTATCACAAACTCTAT<br>AAAGTATTAGAATGTGTATTGCGATGTAACGCATCTATATACAAC<br>TTTCAGCAACGGATCTCTTGGCTCTCGCATCGATGAAGAACGCAG<br>CGAAATGCGATAAGTAATGTGAATTGCAGAATTCAGTGAATCAT<br>CGAATCTTTGAACGCACCTTGCGCTCCTTGGTATTCCGAGGAGCA<br>TGCCTGTTTGAGTGTCATGAAATCTTCAACCTACAAGCCTTTGCG<br>GTTTGTAGGCTTGGACTTGGAGGCTTGTCGGCCCTTTGTCGGTCG<br>GCTCCTCTTAAATGCATTAGCTTGATTCCTTGCGGATCGGCTCTCG<br>GTGTGATAATGTCTAGCCGCGACCGTGAAGCGTTTGGCGAGCTT<br>CTAACCGTCTTCGCTTGAAGACAGCTTTATGACCTCTGACCTCAA<br>ATCAGGTAGACTACCCGCTGAACTTAAGCATATCAATAAGCGGA<br>AGGAAAAAAATCGAGTTCTGACTGGGTTGTAGCTGGCCTTCCGA<br>GGCACGTGCACGCCTGCTCATCACTCTACACCTGTGCACTTACTG<br>TGGGTTTCAGATCTGTGAAGCGTGCCCCTTGCGGGGCTTCTGGA<br>AGCCGTCGTGCTGCGTTTATCCAGACTCTATAAAGTATTAGAATG<br>TGTATTGCGATGTAACGCATCTATATACAACTTTCACACGAAGCT<br>TTGGGCTCTCATCGGATAAAGAAAGCACCCAAATGCGATAAGGT<br>AATGTGAAATTGCAGAAATTCAGTGGAATCATCAAATCTTTGAAA	860.64	97.79%

14	*Irpex lacteus* (Fr.) Fr.	076-18	TCCAAGTTCCAAGCCCCGGTTAAACCTTTCATTAACAAAAATTTTA<br>GGTGAGAATACCCCTGAGACTTCAAAACCGGCATATTTCCTTGGA<br>ATACCCAGGAGGCCAGGGTTACAAAGTTTTGATGATTCCACTGA<br>ATTTCTGCAATTCCACATTACTTTATCGCATTTCGTGCGTTTTTAAT<br>CGATGCAGAGCCCAAAGTTCCGTTGTAAAGTTGTATATAAAATGT<br>GTTATACCCCGTTGACTTTTCTATAAATGAAGCTTGGTAGTAAAA<br>CATAAGAAAGAAAAAAGGCTTGTTCAACCGAAGACCTCTCGGAG<br>TTCCTGGAAGCTTTCCACCATTTTTTTCTTCTTACATAAAGTGCAC<br>AGAGGTTAAGAGGGTTGACCCGGCGTGCACATGCCCCGTGAGA<br>GGCCAGCTACAACCCGTTCAAAACTCGATAATTAACCCTTCTTTCC<br>GTAGGGGTGACTGCGGAAGGATCATTATCGAGTTTTGAACGGGT<br>TGTAGCTGGCCTCTCACGGGGCATGTGCACGCCTGGCTCATCCAC<br>TCTTAACCTCTGTGCACTTTATGTAAGAGAAAAAAATGGTGGAAG<br>CTTCCAGGATCTCGCGAGAGGTCTTCGGTTGAACAAGCCGTTTTT<br>CTTTCTTATGTTTTACTACAAACGCTTCAGTTATAGAATGTCAACT<br>GTGTATAACACATTTATATACAACTTTCAGCAACGGATCTCTTGG<br>CTCTCGCATCGATGAAGAACGCAGCGAAATGCGATAAGTAATGT<br>GAATTGCAGAATTCAGTGAATCATCGAATCTTTGAACGCACCTTG<br>CACTCCTTGGTATTCCGAGGAGTATGCCTGTTTGAGTCTCATGGT<br>ATTCTCAACCCCTAAATTTTTGTAATGAAGGTTTAGCGGGCTTGG<br>ACTTGGAGGTTGTGTCGGCCCTTGTCGGTCGACTCCTCTGAAATG<br>CATTAGCGTGAATCTTACGGATCGCCTTCAGTGTGATAATTATCT<br>GCGCTGTGGTGTTGAAGTATTTATGGTGTTCATGCTTCGAACCGT<br>CTCCTTGCCGAGACAATCATTTGACAATCTGAGCTCAAATCAGGT<br>AGGACCTACCCGCTGAACTTAAGCATATCAATAAGCCGGAGGAA<br>AAGGAAATTATCGAGTTTTGAACGGGTTGTAGCTGGCCTCTCAC<br>GGGGCATGTGCACGCCGGCTCATCACTCTTAACCTCTGTGCACTT<br>TATGTAAGAGAAAAAAAATGGTGGAAGCTTCCAGAATCTCCGAG<br>AAGGTCTTTGGTTGAACAAGCCTTTTTCTTTCTTATGTTTTACTAC<br>AAACGCTTCATTTATAGAAAGGTCAACGTGGTATAACCATTTTAT<br>ATACACTTTCACACGGATCTCTGGGCTCTGCATCAATAAAGAAAC<br>CACCCAAATGCGATAAGTAATGTGAAATTGCAGAAATTCATTGG<br>AATCATCAAAACTTTGAACCTTGCATCCTGGGTATTCCAGGAAGT<br>ATGCTGGTTTGAGTCTCAGGGGTATTCTACCCAAATTTTGTTAAT<br>GAAAGGTTTAACGGGCCTTGGAACTTG	993.77	91.89%
15	*Irpiciporus pachyodon* (Pers.) Kotl. & Pouzar	175-19	CCGTACGTGAACCTGCGGAAGGATCATTATGAATTTTATGAAGG<br>TTGTAGCTGGCCTCAATCGGGGCATGTGCACGCCTTGCTCATTCC<br>AACCTTTTACACCTCTGTGCACTTTACATAGGCTTGGTTTGGGCTG<br>TCCTTTATTGGATGGCTGGCCGGCCTATGCATCTTACAAACGCTT<br>CAGTTTTAGAATGTCATCTGCGTATAACGCAACTATATACAACTTT<br>CAGCAACGGATCTCTTGGCTCTCGCATCGATGAAGAACGCAGCG<br>AAATGCGATAAGTAATGTGAATTGCAGAATTCAGTGAATCATCG<br>AATCTTTGAATGCACCTTGCGCCTCCCGGTATTCCGGGAGGCATG<br>CCTGTTTGAGTGTCATGGTATTCTCAATACCCTAAATCTTTGCGGA<br>TGAGGGTGTATTGGACTTGGAGGTTTTTCTTTGCTGGCTTCATTG<br>TCGGCTCCTCTGAAATACATTAGTGCAGATATTGCTGTTACTCTTC<br>AGTGTGATAATTGTCTACGCTGCAGATGTATGGTGAATTGAAGTC<br>TGTGCTTCTAATTGTCKTTGGACAATTTTTGAAATTCGACCTCAAA<br>TCAGGCAGGACTACCCGCTGAACTTAAGCATATCAATAAGCCGG<br>AAAGAATCAATAATGAATTTTATGACAGGGTTGTAGCTGGCCTCA<br>ATCGGGGCATGTGCACCCTGCTCATTCCACCTTTTACCCTCTGTGC<br>ACTTTACATAGGCCTGGTTTGGGCTGTCCTTTATTGGATGGCTGG<br>CCGGCTATGCATCTTAAAACGCTTCATTTTTAGAATGTCATCTGCT<br>TATAAGCACTATATCCACTTTCGCAACGGAACTCTGGGCTCTGCA<br>TCCATAAAAAAGCACCGAAATGCGATAAGTAATGTGAAATTGCA<br>GAAATTCCTGAAATCTCAAACTTTTAATGCCTGGCGCTCGGTATT<br>CGGGAGGCAGG	886.55	97.28%

16	*Lenzites betulinus* (L.) Fr.	088-19	GTAGGTGAACCTGCGGAAGGATCATTAACGAGTTTTGAAAGGG<br>GGTTGTAGCTGGCCTTCCGAGGCATGTGCACGCCCTGCTCATCCC<br>ACTCTACACCTGTGCACTTACTGTAGGTCGGCGTGGGTTTCTAGC<br>CTCGGGTTTGAAGCATTCTGCTGGCCTATGTACATTTATAAACAC<br>TTTAAAGTAACAGAATGTAAACGCGTCTAACGCATTTTAATACAA<br>CTTTCAGCAACGGATCTCTTGGCTCTCGCATCGATGAAGAACGCA<br>GCGAAATGCGATAAGTAATGTGAATTGCAGAATTCAGTGAATCA<br>TCGAATCTTTGAACGCACCTTGCGCTCCTTGGTATTCCGAGGAGC<br>ATGCCTGTTTGAGTGTCATGGAATTCTCAACCCATAAATCCTTGT<br>GATGTATGGGCTTGGATTTGGAGGCTTGCTGGTCCCTTTCGGGA<br>TCGGCTCCTCTTGAATATATTAGCTTGATTCCGTGCGGATCGGCT<br>CTCAGTGTGATAATTATCTACGCTGTGACCGTGAAGCGTTTGGCG<br>AGCTTCTAACCGTCCTTTTTGGACAAACCTTATGACATCTGACCTC<br>AAATCAGTAGACACCCGCTGAACTTAAGCATATCAATAAGCGGA<br>GGAAGTTCATTAACGAGTTTTGAAAGGGGTTGTAGCTGGCCTTC<br>CGAGGCATGTGCACGCCTGCTCATCCACTCTACACCTGTGCACTT<br>ACTGTAAGTCGGCGTGGGTTTCTAGCCTCTGGGTTTGAACCATTC<br>CTCTGGCCAATGCCCATTTATAACACCTTTAAGGTAACAGAATGA<br>TAAACGCCTCTAACGCTTTTAATACATCTTCCACAAGGGATCTTTG<br>GGCTCCACCCCTTAAAAAAACACCGAAAATGCATTAAGTAAAGG<br>TGAATGCACAAATTCAGGAAACCTCAAATCTTTAAACCCCTGCCC<br>CCTGGTTATTCCAAGAAACAAGCGGTTTGAGGTGTCCGGGAAAT<br>TCCCCAAAA	871.73	97.55%
17	*Polyporus alveolaris* (DC.) Bondartsev & Singer	096-19	GCTTTATGAATTTGGAGGGGTCGATGTGAGCGCCGAACAGTCCT<br>TCCCAAGTCCCAGGCTTAAAGACCCCAGAAAGGGTTTATAAGTG<br>AGGATTTTCTGAACACTCAAAACGGGTGCTTTCTGGAATACCCAA<br>GAGCCCAGGGGTTAAAAGTTTGAGTGATTCAAGACTTCTGCTATT<br>TCACATTAACTGACCGCATTGCGTGCTTTTTTAATGGCTGCAGAG<br>CCCAAAGTTTCCTCGCGACAGTGTATATAGATGGTGATGGCCATA<br>CACTTTCTTTTATTGAAGCGTGGTATATAAAGACAGGTAGGGGCA<br>AAAAGCCCCACGATACCCGACTATAAGTGCACGTGTAGAGGGAT<br>TGAGCAGGCTGCACTTGCCCCGAACGGCTAGCTACAACCCCGAC<br>TCCTATGGAGAAACTTACCCCTTCCGTAGGTGAACCTGCGGAAG<br>GATCATTATCGAGTTTTGATCGGGGTTGTAGCTGGCCTTTCGGGG<br>CATGTGCACGCCCTGCTCAATCCACTCTACACCTGTGCACTTATTG<br>TGGGTTTCGGGGGGCTTTTTGCCACTCGTACCTATGTTTATTTACA<br>AACGCTTCAGTAAAAGAATGTGTATTGCGATAACGCATCTATATA<br>CAACTTTCAGCAACGGATCTCTTGGCTCTCGCATCGATGAAGAAC<br>GCAGCGAAATGCGATAAGTAATGTGAATTGCAGAATTCAGTGAA<br>TCATCGAATCTTTGAACGCACCTTGCGCTCCTTGGTATTCCGAGG<br>AGCACGCCTGTTTGAGTGTCATGAAATCATCAACTTATAAGCCTT<br>TCATGGGTCTGTAAGCTTGGACTTGGAGGCCTGTCGGCGGTCAA<br>CGTCGGCTCCTCTCAAATGTATTAGCTTGGTTCCTTGCGGATCGG<br>CTCCCGGTGTGATAATGTCTACGCTGTGACCGTGAAGCGTTTGGC<br>GAGCTTCTAATCGTCTCGTGAGAGACAGCTTCATTGACTCTGACC<br>TCAAATCAGGCGGGACACCCGTAACTTAAGCATATCAATAATTCG<br>GAGAAAAAAAAGATCGTTCCGAGAGGAGTCCGGGTTGGAGGGT<br>GGCCTTTCGGGGCACGTGCCGCCTGCTCAATCCCTCTACCCTGTG<br>CACTTATAGTGGGTTTCCGGGGGGCTTTTTGCCCTCGTACCTATG<br>TTTATATACAAACGCTTCGTAAAAGAAAGTGTTATTGCGATAACG<br>CATCTATTTATCACTCTCGCAACGGAGCTTTTGGCTCTGCACCCAT<br>CAAAAACTCACCCCACTGCGATTAGTTAATGTGCAATTGCATAAA<br>TTCCTGCAATCTCAAAACTTTTAACCCCTGGGCTCTTGCTATTCTA<br>GAATCACCCGTTTGAGTGTCAGAAAA	778.22	94.51%

18	*Stereum hirsutum* (Willd.) Pers.	073-18	GAGGGCAAAGTTCGATGAAAAGCTGTCCTTTCGGACGGTTAGAA<br>AGCGCGTCTCCACCGAAGCAAGCACACCACAGCGTAGATAATTA<br>TCACACCGAGGCCCACGTCGCAACAAGACGCACTAATGCATTTG<br>AGAGGAGCCGAGCGGTGAAGCCCGGCAAAGCCTCCAAGTCCAA<br>TCCACTACGTTCACAAAAGTGAAGAGGGTTGAGAATTTCACGAC<br>ACTCAAACAGGTGTGCCCTTCGGAATACCAAAGGGCGCAAGGTG<br>CGTTCAAAGATTCGATGATTCACTGAATTCTGCAATTCACATTACT<br>TATCGCATTTCGCTGCGTTCTTCATCGATGCGAGAGCCAAGAGAT<br>CCGTTGTTGAAAGTTGTATTAGATGCGTTTTATTACATCGAGTAG<br>ACATTCTTAAGACATACAAAGGGTGTGTAAAGGGACGCGAGCCT<br>CCTCCAAATAACCAAAAAACACCCGCGAAAGTGCGCACGTGTGT<br>GTGTGGAATAGAAAGGAGAGCGCGCATGTGGCGTTTATAGGCG<br>ACATATACCTCTCCACACATATATTTCATAAGAGATCTCCGCACGT<br>GCACCCCCACGAAGAG	727.50	94.52%
19	*Terana caerulea* (Schrad. ex Lam.) Kuntze	177-19	GGCCAGCCGCGACCTTCCAAGGTTCCCAAGGCTTTCCAAACCAAT<br>AAAAGGGAAGGTGGAAGGTCCCCTGACCACTCCAAACCGGCTGC<br>TTCCTGGAAATACCCAAGAGCGCGAGTGCGTGCAGAGTTTGGTG<br>ATTTACAGGAATTTTGTGCAATTTCACCATTAACTGATCGCACTTC<br>GGTGCTTTCTTAATCGATGCAGAGCCCCAAAGATCCGTTGCGAAA<br>GTGGTATATAATGCTTATAAGCCAGATGACTTTTCTATAACTGAA<br>GCGTGTGTGAGAAGACATAGAGTGCTCGCTAGCGCACCGAAGC<br>GCAGCCGTTCACCTTTCTACAATGAGGCACAGAGGGGAGAGGG<br>GTGAGCCAGGGGCACATGCCCCGGAGAGGCCAGCGACAACCCT<br>TTCAAAACCCCATAATTGATCCTTCCGTAAGGGTGACCTGCGGAA<br>GGTCATTATCAGTTTGACGGGTTGTCGCTGGCCTCCTCGCGGGG<br>CATGTGCACACCTGGCTCATCCACTCTCCACCTCTGTGCACTCATT<br>GTAGAATGGTGAACGGCTGCGCTTCGGTGCGGCTAGAAGCCACT<br>CTATGTCTTATTACACACGCTTCAGTTATAGAATGTCATCTTGCGT<br>ATAACGCATTATATACAACTTTCAGCAACGGATCTCTTGGCTCTC<br>GCATCGATGAAGAACGCAGCGAAATGCGATAAGTAATGTGAATT<br>GCAGAATTCAGTGAATCATCGAATCTTTGAACGCACCTTGCGCTC<br>CTTGGTATTCCGAGGAGCACGCCTGTTTGAGTGTCATGGACTTCT<br>CAACCTTCCACTTTATTGTTGGAGGCTTGGACTTGGAGGTCGTGC<br>TGGCTCTCGTAGTCGGCTCCTCTGAAATGTATTAGCGTGAACGTT<br>ACGGATCGCTTCAGTGTGATAATTATCTGCGCTGTTGTGTTGAAG<br>TATAGTCAGCTTGCGCTTCTAATCGTCCTCACGGACACTATATTGA<br>CAATCTGACCTCAAATCAGGCGGGACTACCCGCTGAACTTAAGC<br>ATATCAATAAGCCGGAGGAAATCAATATCGAGTTTTGAACGGGT<br>TGTCGCTGGCCTCTCCGGGGCATGTGCACACCTGGCTCATCCACT<br>CTCCCCTCTGTGCACTCATTGTAGAATGGTGAACGGCTGCGCTTC<br>GGTGCGGCTAGAAGCACTCTATGTCTTCTTACACACGCTTCATTT<br>ATAGAATGTCATCTTGCTTATAAGCATTATATACAACTTTCGCAAC<br>GGATCTCTTGGCTCTGCATCGATGAAGAAGCACCGAAATGCGAT<br>AAGTTAATGTGAAATTGCAGAAATTCATGGAAATCTCAAATCTCT<br>GAAGCCCTTGGCCTCTTGGTTATTCCAGGAGCAGCCGTTTTGAGT<br>GTCAGGGACTTCTCCCTTCCCTTTATTGGTTGGAAGGCTTGGGAC<br>TTGGAAGGTCGTGCTGGGCTCCTAAATCGCCCCCTGAAAATGGT<br>ATTTAGCGTGGAAGCTTTACGGAATCCTTCCTGTGGATAAATTAA<br>ACTGGCCCGGTGGGGTTTGAAGATAAAAATCAAACCTG	911.35	95.06%
20	*Trametes hirsuta* (Wulfen) Pilát	067-18	GTTGTTGCTGGCCTTCCGAGGCATGTGCCCTGCTCATCCACTCTA<br>CATGTAGGTTGGCGTGGGTTTCTAGCCTCCGGCTGAAGCATTCTG<br>CCGGCCTATGTACACTACAAACTCTTAAAGTATCAGAATGTAAAC<br>GCGTCTAACGCATCTTAATACAACTTTCAGCAACGGATCTCTTGG<br>CTCTCGCATCGATGAAGAACGCAGCGAAATGCGATAAGTAATGT<br>GAATTGCAGAATTCAGTGAATCATCGAATCTTTGAACGCACCTTG<br>CGCTCCTTGGTATTCCGAGGAGCATGCCTGTTTGAGTGTCATGAA<br>ATTCTCAACCCATAAATCCTTGTGATCTATGGGCATGGAT	456.47	98.79

21	*Trametes suaveolens* (L.) Fr.	070-18	CCTGCGGAAGGATCATTAACGAGTTTTGAAACGGGTTGTGCTGG<br>CCTTCCGAGGCATGTGCACGCCCTGCTCATCCACTCTACACCTGT<br>GCACCTACTGTAGGTTGGCGTGGGCTCCTTCGCGGGAGCGTTCT<br>GCCGGCCTATGTATATTACAAACCCCTTAAAGAAACAGAATGTAA<br>ACGCGTCTAACGCATCTTAATACAACTTTTAGCAACGGATCTCTT<br>GGCTCTCGCATCGATGAAGAACGCAGCGAAATGCGATAAGTAAT<br>GTGAATTGCAGAATTCAGTGAATCATCGAATCTTTGAACGCACCT<br>TGCGCTCCTTGGTATTCCGAGGAGCATGCCTGTTTGAGTGTCATG<br>AAATTCTCAACCCGTAAATCCTTGTGATCTACGGGCTTGGACTTG<br>GAGGCTTGCTGGCCCTTGCGGTCGGCTCCTCTTGAATGCATTAGC<br>TTGATTCCGTATGGATCGGCTCTCAGTGTGATAATTGTCTACGCT<br>GTGACCGTGAAGTGTTTTGGCGAGCTTCTAACCGTCTGTTAGGAC<br>AACTTTTTAACATCTGACCTCAAATCAGGTAGGACTACCCGCTGA<br>ACTTAACCATATCAGTAA	874.90	99.82%
